# Genetic contribution to multiple sclerosis risk among Ashkenazi Jews

**DOI:** 10.1186/s12881-015-0201-2

**Published:** 2015-07-28

**Authors:** Pouya Khankhanian, Takuya Matsushita, Lohith Madireddy, Antoine Lizée, Lennox Din, Jayaji M Moré, Pierre-Antoine Gourraud, Stephen L Hauser, Sergio E Baranzini, Jorge R Oksenberg

**Affiliations:** Department of Neurology, University of California, San Francisco, 675 Nelson Rising Lane, San Francisco, CA 94158 USA; Current address: Graduate School of Medical Sciences, Kyushu University School of Medicine, 3-1-1, Maidashi, Higashi-ku, Fukuoka City 812-8582 Japan

**Keywords:** Multiple sclerosis, Ashkenazi jews, Genome-wide association study, Population genetics

## Abstract

**Background:**

Multiple sclerosis (MS) is an autoimmune disease of the central nervous system, with a strong genetic component. Over 100 genetic loci have been implicated in susceptibility to MS in European populations, the most prominent being the 15:01 allele of the *HLA-DRB1* gene. The prevalence of MS is high in European populations including those of Ashkenazi origin, and low in African and Asian populations including those of Jewish origin.

**Methods:**

Here we identified and extracted a total of 213 Ashkenazi MS cases and 546 ethnically matched healthy control individuals from two previous genome-wide case-control association analyses, and 72 trios (affected proband and two unaffected parents) from a previous genome-wide transmission disequilibrium association study, using genetic data to define Ashkenazi. We compared the pattern of genetic risk between Ashkenazi and non-Ashkenazi Europeans. We also sought to identify novel Ashkenazi-specific risk loci by performing association tests on the subset of Ashkenazi cases, controls, probands, and parents from each study.

**Results:**

The HLA-DRB1*15:01 allele and the non-HLA risk alleles were present at relatively low frequencies among Ashkenazi and explained a smaller fraction of the population-level risk when compared to non-Ashkenazi Europeans. Alternative HLA susceptibility alleles were identified in an Ashkenazi-only association study, including HLA-A*68:02 and one or both genes in the HLA-B*38:01-HLA-C*12:03 haplotype. The genome-wide screen in Ashkenazi did not reveal any loci associated with MS risk.

**Conclusion:**

These results suggest that genetic susceptibility to MS in Ashkenazi Jews has not been as well established as that of non-Ashkenazi Europeans. This implies value in studying large well-characterized Ashkenazi populations to accelerate gene discovery in complex genetic diseases.

**Electronic supplementary material:**

The online version of this article (doi:10.1186/s12881-015-0201-2) contains supplementary material, which is available to authorized users.

## Background

Multiple sclerosis (MS) is an autoimmune disease with central nervous system pathology [1] and the most common cause of non-traumatic neurological disability in young adults, affecting approximately 2.5 million people worldwide. The global burden of MS has increased over the past century but retains the well-known influence of gender, latitude, and ancestry on risk. This is reflected in the relatively high incidence in some population groups (particularly those of European origin) compared with others (African and Asian groups) [2, 3]. Likewise, high frequency rates are found in Scandinavia, Iceland, the British Isles and North America (about 1–2 in 1,000), but lower frequencies are observed in the Mediterranean Basin (0.5 in 1000).

There are notable exceptions to the European prevalence gradient, such as in Sardinia where the prevalence of MS is among the highest in the world (1.4/1,000) [4]. Similarly, Ashkenazi Jews in Israel (and the Diaspora) are at high risk for developing MS [5]. The distinctive population histories of Sardinians and Ashkenazi Jews include founder effects, admixture, and bottlenecks, suggesting that unique genetic signatures underlie their differential susceptibility. Unraveling these profiles may provide important insights into the genetics of MS and interactions with non-genetic factors.

Extensive empirical evidence confirms that genetic variation is an important determinant of MS risk. Multiple genome-wide association studies (GWAS) have been completed and reported, including a multi-center effort with nearly ten thousand cases [6]. The classic *HLA-DRB1* risk locus within the MHC, specifically the HLA-DRB1*15:01 allele stood out in all GWAS with remarkably strong statistical significance. In addition, 110 non-MHC variants were found to be associated with disease susceptibility (Additional file 1: Table S1) [7]. As expected, each identified variant conferred only modest odds ratios. These studies have focused on datasets ascertained in Europe, Australia and North America and included affected Ashkenazi individuals. Jewish populations in the Diaspora can be grouped into distinct genetic clades shaped by admixture with local populations and social and cultural forces. Nevertheless, they all maintain the ancestral Eastern Mediterranean and Middle Eastern genomic identity, which contrasts significantly with central and northern European populations [8–12]. Here we seek to clarify the genetic characteristics of MS in Ashkenazi Jews.

Previous studies reported the HLA-DRB1*15:01 association with MS in Ashkenazi Jews living in Israel, albeit with reduced odds ratios compared to Europeans [13]. The HLA-DRB1*13:03 allele was the strongest genetic biomarker of risk in a study of non-Ashkenazi Israelis [13] but was not confirmed in Ashkenazi Israelis [13]. In our study, we use genomic signatures to identify and extract Ashkenazi Jewish individuals from three SNP-chip-based genome wide studies: a transmission disequilibrium (TD) study and two case control studies. We next illustrate the genetic relationship of the Ashkenazi subsets to the other European sub-populations. We then compare allelic risk at *HLA-DRB1* and polygenic risk across the genome between Ashkenazi Jews and non-Jewish western Europeans, based on previously suggested risk alleles. To identify MS susceptibility alleles that may be specific to the Ashkenazi population, we perform case–control and TD tests for SNPs across the genome and at classical HLA alleles in the Ashkenazi subset.

## Results

### Overview

We started by defining Ashkenazi subjects from three previous multiple sclerosis studies: two genome-wide case–control association studies (GENEMSA and WTCCC2) and one genome-wide transmission disequilibrium (TD) trio study (IMSGC). We describe the position of Ashkenazi Europeans within the genetic landscape composed of other European populations available in the WTCCC2 dataset. We compare selected clinical characteristics of Ashkenazi to non-Ashkenazi Europeans using available data in the GENEMSA dataset. We then proceed to examine the MS genetic risk burden in Ashkenazi using previously identified MS susceptibility variants. Due to the unique properties of the HLA risk compared to the genome-wide risk, we assess the HLA risk before evaluating the genome-wide genetic burden at 110 non-HLA MS susceptibility SNPs en masse (using a 110-SNP MS genetic burden score, termed the MSGB). We compare Ashkenazi cases to Ashkenazi controls to determine whether previously identified risk alleles apply to the Ashkenazi population. We compare MS risk attributable to the previously identified genetic susceptibility loci between Ashkenazi and non-Ashkenazi Europeans.

Finally, we seek to identify Ashkenazi-specific risk loci. We perform case–control analyses in Ashkenazi subsets of the GENEMSA and WTCCC2 studies and perform a TD test of the Ashkenazi trios of the IMSGC study.

### Defining the Ashkenazi subsets of previous studies

**Fig 1:**
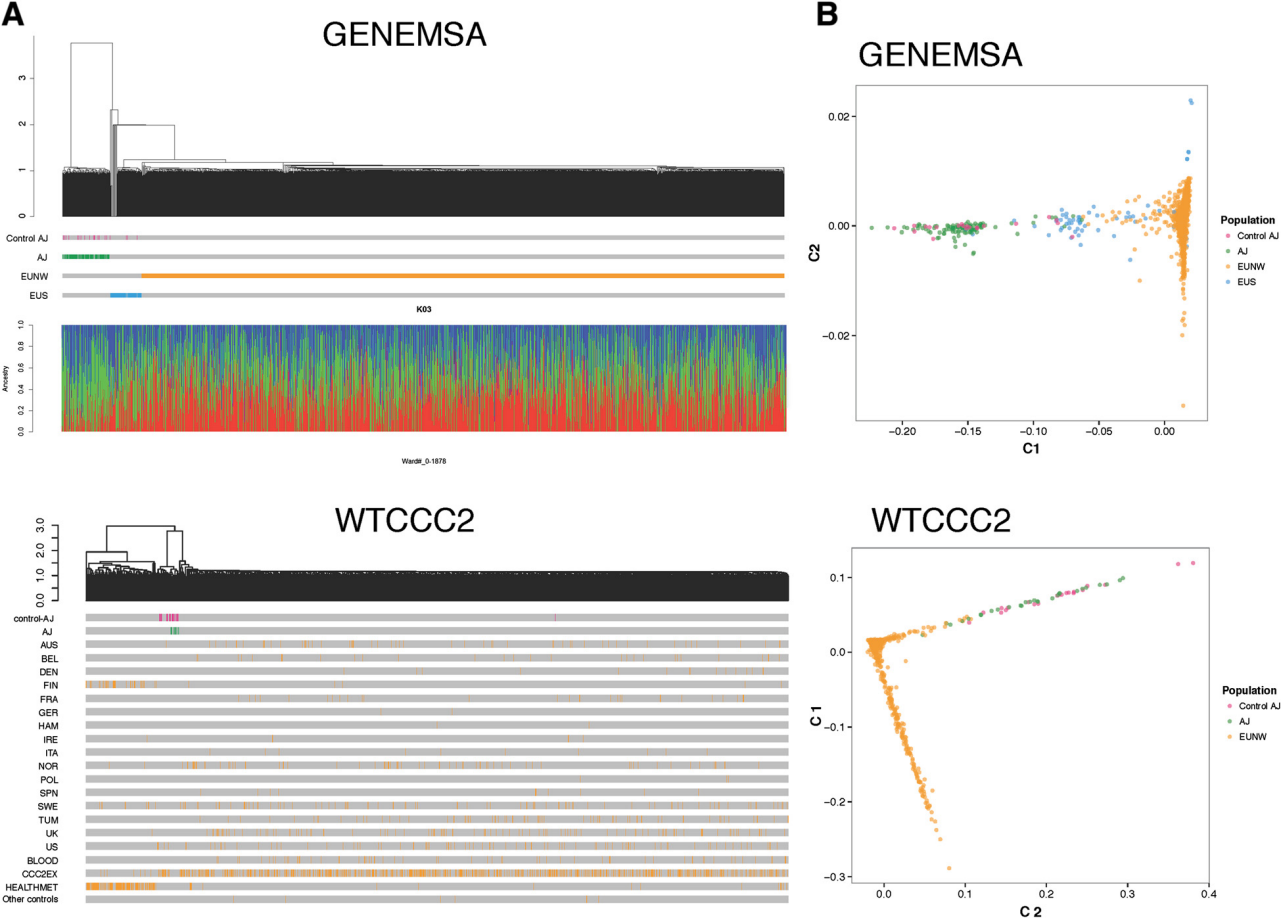
Hierarchical clustering and multi-dimensional scaling of individuals. **a**: Hierarchical clustering using genome-wide IBD in GENEMSA and the WTCCC2 dataset. The dendrogram demonstrates relationship between individuals and the lower bars designate assigned population of each individual. Control Ashkenazi Jews (AJ) corresponds to ethnically well-characterized Ashkenazi controls [9]. The lower panel shows individual ancestry and admixture proportions with K = 3. **b**: Multidimensional scaling of genome-wide IBD in GENEMSA and WTCCC2 dataset (pink = previously well defined AJ controls [9], green = newly defined AJ cases and controls, yellow = EUNW, blue = EUS)

To extract individuals from case–control studies (GENEMSA and WTCCC2) who had genetic similarity to a well-defined Ashkenazi genetic reference dataset, we built a hierarchy of clusters based on genome-wide IBD distances. In the GENEMSA study, hierarchical clustering revealed a large cluster of northern and western European individuals (EUNW), a small cluster of eastern European Ashkenazi Jews, and a small cluster of southern Europeans (EUS). In the WTCCC2 study, there was a large cluster of EUNW, a small cluster of European Ashkenazi Jews, and a small cluster of Finnish samples. Based on the hierarchical clustering, 97 Ashkenazi Jews and 1,541 EUNW in GENEMSA and 662 Ashkenazi Jews and 26,487 EUNW in WTCCC2 were identified (Fig. 1a). Multidimensional scaling of genome-wide IBD distances (Fig. 1b) revealed a large main cluster of EUNW and a smaller outlying cluster of the well-characterized Ashkenazi controls in both datasets. Structure-like analysis using FRAPPE [14] (a maximum likelihood method to infer the genetic ancestry of each individual, where the individuals are assumed to have originated from K ancestral clusters) with K = 3 also demarcated the cluster of Ashkenazi individuals (Fig. 1a).

To extract individuals of Ashkenazi origin from the IMSGC trio study, multi-dimensional scaling was performed as above. In this case, the first principal component (PC1) was used to define Ashkenazi individuals (Additional file 5). For each trio, the value of PC1 for the proband was approximately the mean of the value of PC1 for the parents. Higher values of PC1 were defined as Ashkenazi Jews and lower values were defined as EUNW. For the purpose of the transmission disequilibrium test (see meta-analysis section below), 76 Ashkenazi trios were identified, where an Ashkenazi trio is defined as a trio with at least one Ashkenazi parent. Approximately one third (24 of 76) families had one non-Ashkenazi parent, and in those families transmission from either parent was considered for the analysis.

Detailed MS clinical characteristics of the Ashkenazi with respect to Europeans were available for a small proportion of the samples and are reported in the Additional file 2. Some intriguing differences such as significantly lower multiple sclerosis severity score in Ashkenazi, and lower rates of motor weakness and acute transverse myelitis require confirmation with larger datasets.

### Relationship of Ashkenazi to other Europeans

**Fig 2:**
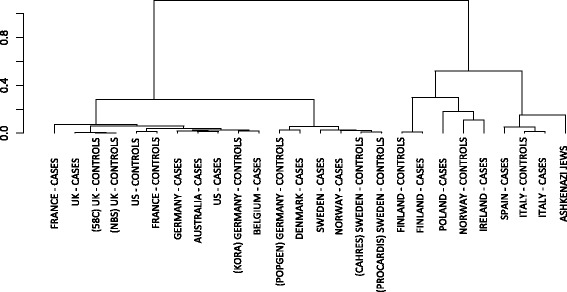
Hierarchical clustering of populations. A hierarchical clustering of cohorts that comprise the WTCCC2 dataset. The y-axis represents genome-wide distance between populations (measured from genome-wide allele frequencies). Ashkenazi Jews cluster with the Spanish cases, the Italian cases, and the Italian controls, as seen on the right. On the left, a large cluster of western European cases and controls from France, Belgium, Germany, Australia, United States, and the United Kingdom. In the center, a cluster of northern European countries includes Sweden, Denmark, Norway, and Germany. Two Finnish populations cluster tightly together. Three groups of relatively small sample size cluster loosely including groups from Poland, Norway, and Ireland. For countries with two sets of controls (UK, Germany, Sweden), the cohort names are provided in parentheses [6]

The WTCCC2 MS dataset was collected in 15 different countries spanning Europe, the US, and Australia. To explore the relationship between Ashkenazi and other populations of European ancestry, hierarchical clustering of data from each country using whole-genome SNP frequencies was performed (Fig. 2). This analysis showed that Ashkenazi Jews grouped with Italian and Spanish populations. The majority of the Ashkenazi study participants came from UK and US populations. Populations from Australia, Belgium, Denmark, France, Germany, Sweden, the UK and the US (excluding the Ashkenazi Jews) formed a larger cluster. The Finnish population was relatively distant from the other European populations.

### LD Decay

**Fig 3:**
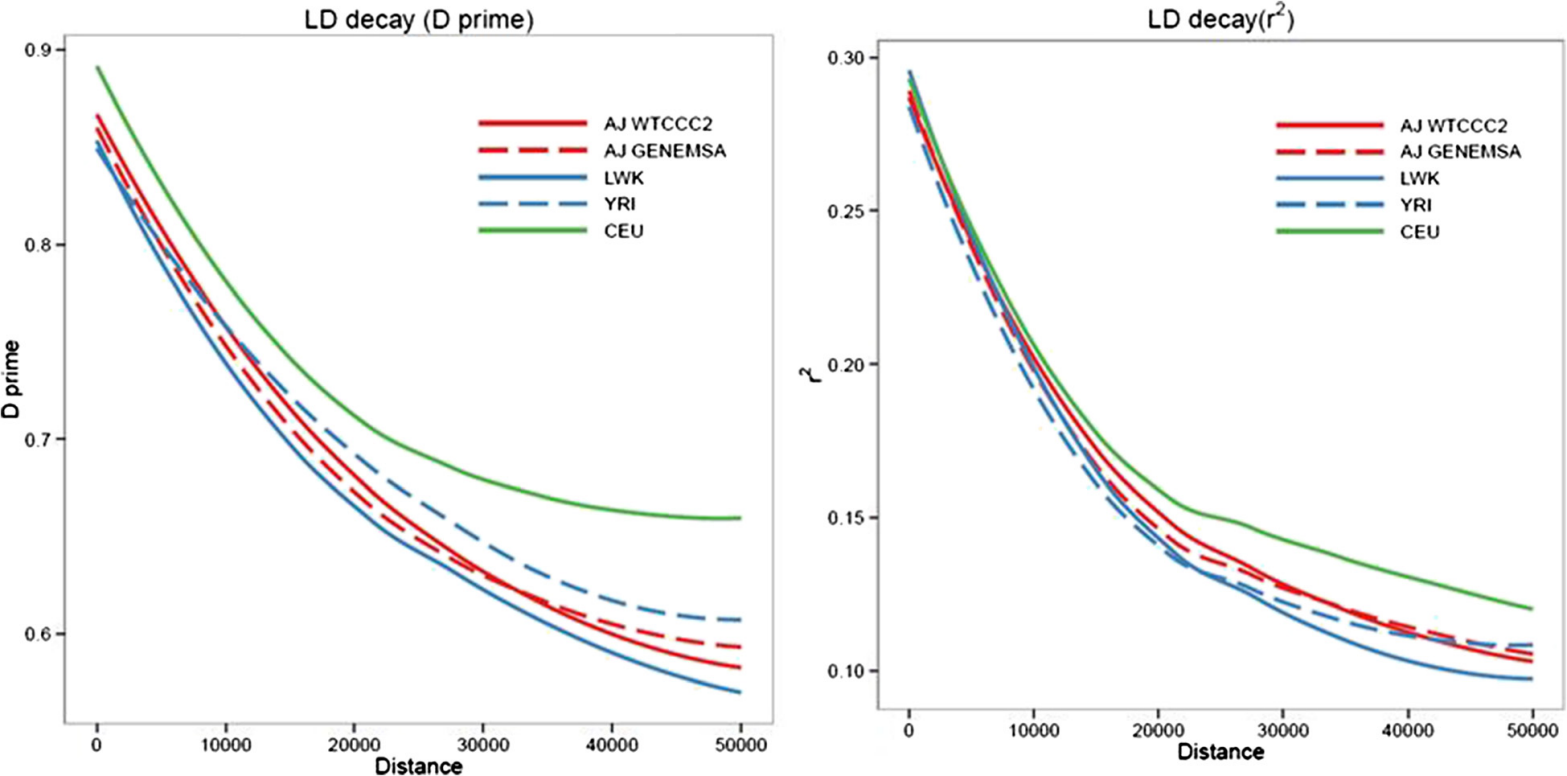
Linkage Disequilibrium. The median D-prime (y-axis, left panel), and median r-squared (y-axis, right panel), as a function of genetic distance in base pairs (x-axis), for six groups of samples. CEU = reference northwestern European samples from Utah. LWK, YRI = reference sets of African samples. AJ-WTCCC2, AJ-GENEMSA = two sets of Ashkenazi samples this study

Two measures of linkage disequilibrium (D-prime and r-squared) were plotted as a function of genetic distance in base pairs for five groups of samples (Fig. 3). The CEU in green is a reference set of northwestern Europeans and the LWK and YRI samples in blue are two reference sets of African samples, all from the 1000 genomes project (www.1000genomes.org). The AJ-WTCCC2 and AJ-GENEMSA in red are two sets of Ashkenazi samples from the current study. The same number of individuals were used for all five groups. We observed that the northwestern European population exhibits greater LD than Ashkenazi and African samples, suggesting that well powered Ashkenazi datasets could add to fine mapping of regions of interests identified in GWAS.

### MS genetic burden of Ashkenazi: HLA

**Fig 4:**
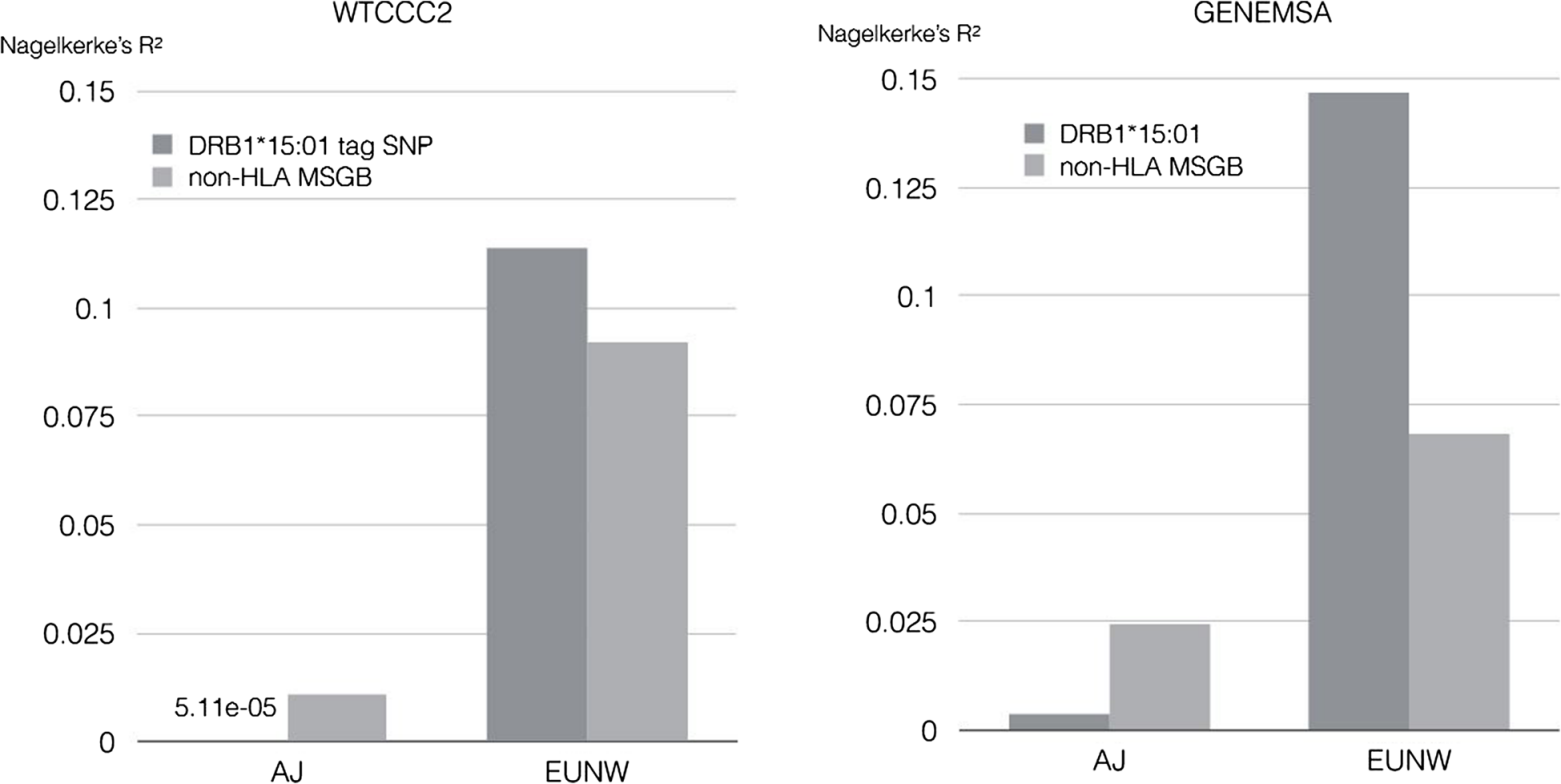
Multiple sclerosis risk attributed to genetics. The Multiple sclerosis risk (Nagelkerke R^2^) attributable to HLA-DRB1*15:01 and the non-HLA MSGB score in the GENEMSA and WTCCC2 datasets

To search for HLA alleles associated with MS risk in Ashkenazi, a case–control association study of imputed HLA alleles was performed on the GENEMSA and WTCCC2 datasets, and a genome-wide transmission disequilibrium test was performed on the IMSGC dataset. The HLA-DRB1*15:01 allele conferred risk in each of the three studies, statistically significant in two of three (Table 1). The HLA-DQB1*06:02 allele, highly linked to HLA-DRB1*15:01, showed a similar pattern of risk. The HLA-DRB1*13:03 allele, previously implicated in an a non-Ashkenazi Israeli MS dataset [13], was found in only 11 total Ashkenazi individuals from the three datasets and showed no evidence of association with disease. The population risk attributable to a variant (Nagelkerke r^2^) depends on the effect size (the odds ratio) and the frequency of the risk variant in cases. While the odds ratio for the HLA-DRB1*15:01 allele in Ashkenazi Jews is similar to that in EUNW (Additional file 4), the HLA-DRB1*15:01 carrier frequency is lower in Ashkenazi (for example, 15.6 % in WTCCC Ashkenazi controls versus 28.1 % in WTCCC EUNW controls) (Additional file 4). It is therefore not surprising that the risk explained by the HLA-DRB1*15:01 allele is lower in Ashkenazi Jews (GENEMSA Nagelkerke r^2^ = 0.009, WTCCC2 r^2^ < 0.0001) than in EUNW (GENEMSA r^2^ = 0.145, WTCCC2 r^2^ = 0.11) (Fig. 4).

**Table 1:** HLA alleles which are associated with MS Risk in Ashkenazi

	WTCCC2		GENEMSA		IMSGC		Meta
Allele	OR	P	OR	P	OR	P	P
*A* ^*^ *68:02*	3.67	0.0043	2.29	0.3361	1.00	1.0000	0.0415
*B* ^*^ *38:01*	0.71	0.3996	0.65	0.4350	0.08	0.0013	0.0103
*C* ^*^ *12:03*	0.74	0.2230	0.66	0.4474	0.14	0.0000	0.0001
*DRB1* ^*^ *15:01*	2.13	0.0072	7.17	0.0728	3.00	0.0455	0.0016
*DQB1* ^*^ *06:02*	2.19	0.0099	NA^a^	0.0325	3.00	0.0047	0.0002

^a^all Ashkenazi carriers of DQB1^*^06:02 were cases

Three other HLA alleles showed nominal level of association (p < 0.05, uncorrected for multiple hypothesis testing) in a meta-analysis of the three datasets. The HLA-A*68:02 showed positive association with MS (p = 0.04), while the HLA-B*38:01 and HLA-C*12:03 alleles showed negative association (p = 0.01 and 0.0001 respectively).

**Fig 5:**
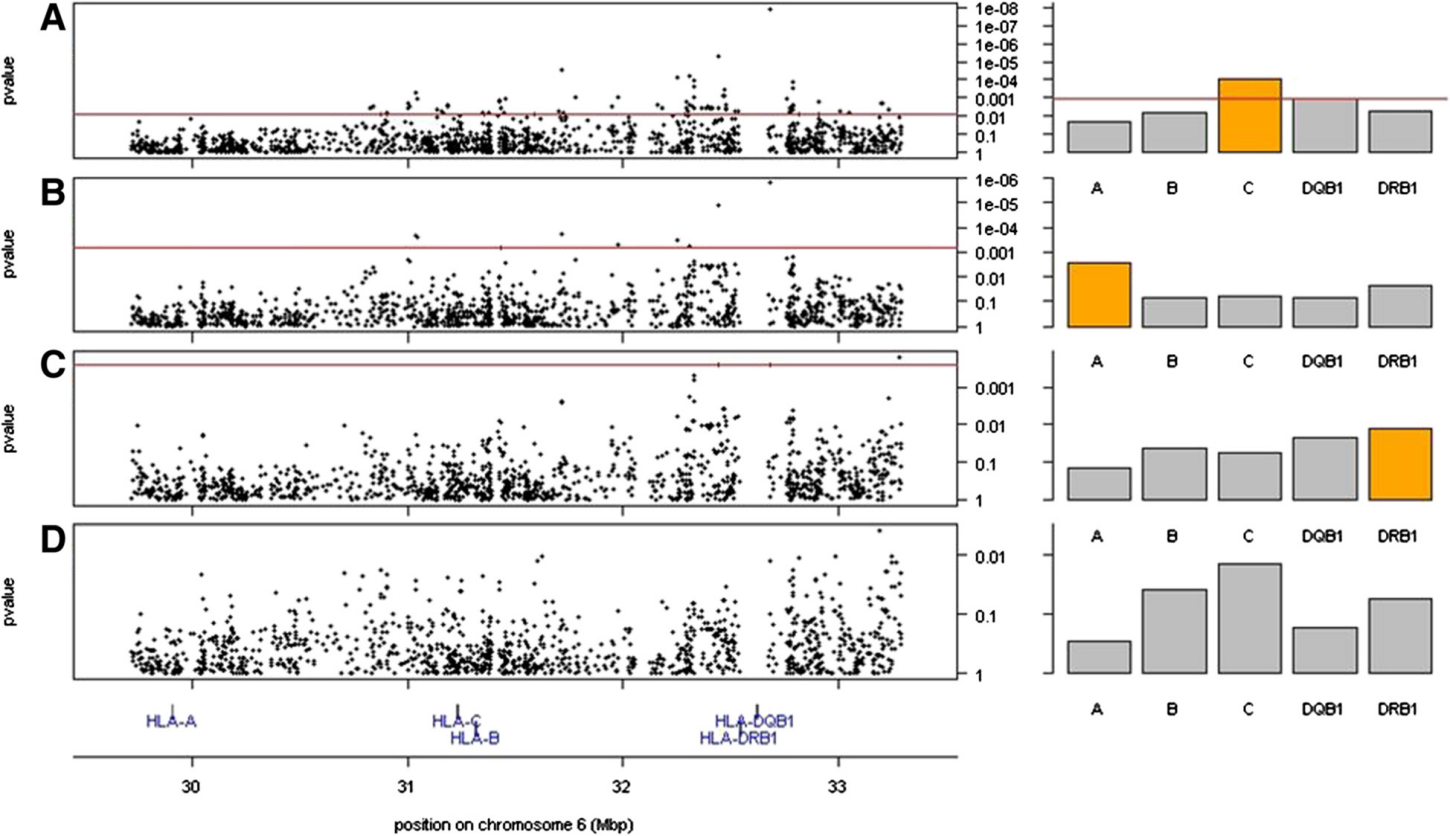
Human Leukocyte Antigen Alleles associated with multiple sclerosis in Ashkenazi Jews. A step-wise conditional analysis was performed to evaluate statistically significant independent HLA effects. Panel **a** left: SNPs from across MHC class I and class II passed the Bonferroni threshold of 0.05 (red line on figure), the top hit coming from class II near *DRB1* and *DQB1*. Panel **a** right: The top significant HLA allele came from the *HLA-C* gene and was the *12:03 allele. Panel **b**: In the second analysis, HLA-C*12:03 was included as a covariate. Here again a handful of SNPs passed the Bonferroni threshold, and the top significant finding was at the *HLA-A* gene, where the *68:02 allele was most significant. Panel **c**: In the third analysis, HLA-C*12:03 and HLA-A*68:02 were both included as covariates. Here 3 SNPs passed the Bonferroni cutoff, and the top finding was the *15:01 allele at *HLA-DRB1*. Panel **d**: In the fourth analysis, the covariates were HLA-C*12:03, HLA-A*68:02, and HLA-DRB1*15:01 and no further statistically significant associations were seen

A step-wise conditional analysis (Fig. 5) was performed to evaluate statistically significant independent HLA effects. In the first analysis (Fig. 5 panel a), SNPs from across MHC class I and class II (panel A, left side) passed the Bonferroni threshold of 0.05 (red line on figure). The first significant HLA allele (most significant p-value), came from the *HLA-C* gene (panel A right side) and was the C*12:03 allele (thought the allele does not pass Bonferroni correction). In the second analysis, HLA-C*12:03 was included as a covariate and we looked for an independent effect (panel B). Here again a handful of SNPs passed the Bonferroni threshold, and the most significant finding was at the *HLA-A* gene, where the A*68:02 allele was most significant. In the third analysis, HLA-C*12:03 and HLA-A*68:02 were both included as covariates (panel C). At this stage, only 3 SNPs passed the Bonferroni cutoff, and the top finding was the HLA-DRB1*15:01 allele. In the fourth and final analysis, the covariates were HLA-C*12:03, HLA-A*68:02, and HLA-DRB1*15:01 (Panel D); no further statistically significant SNP or HLA allele associations were seen. Of note, in each step of the analysis, the top HLA allele was chosen as a covariate for the next step, even if a single SNP may have had higher significance than the HLA allele, and even if the HLA allele did not pass Bonferroni threshold.

### MS genetic burden of Ashkenazi: Genome-wide non-HLA risk

**Fig 6:**
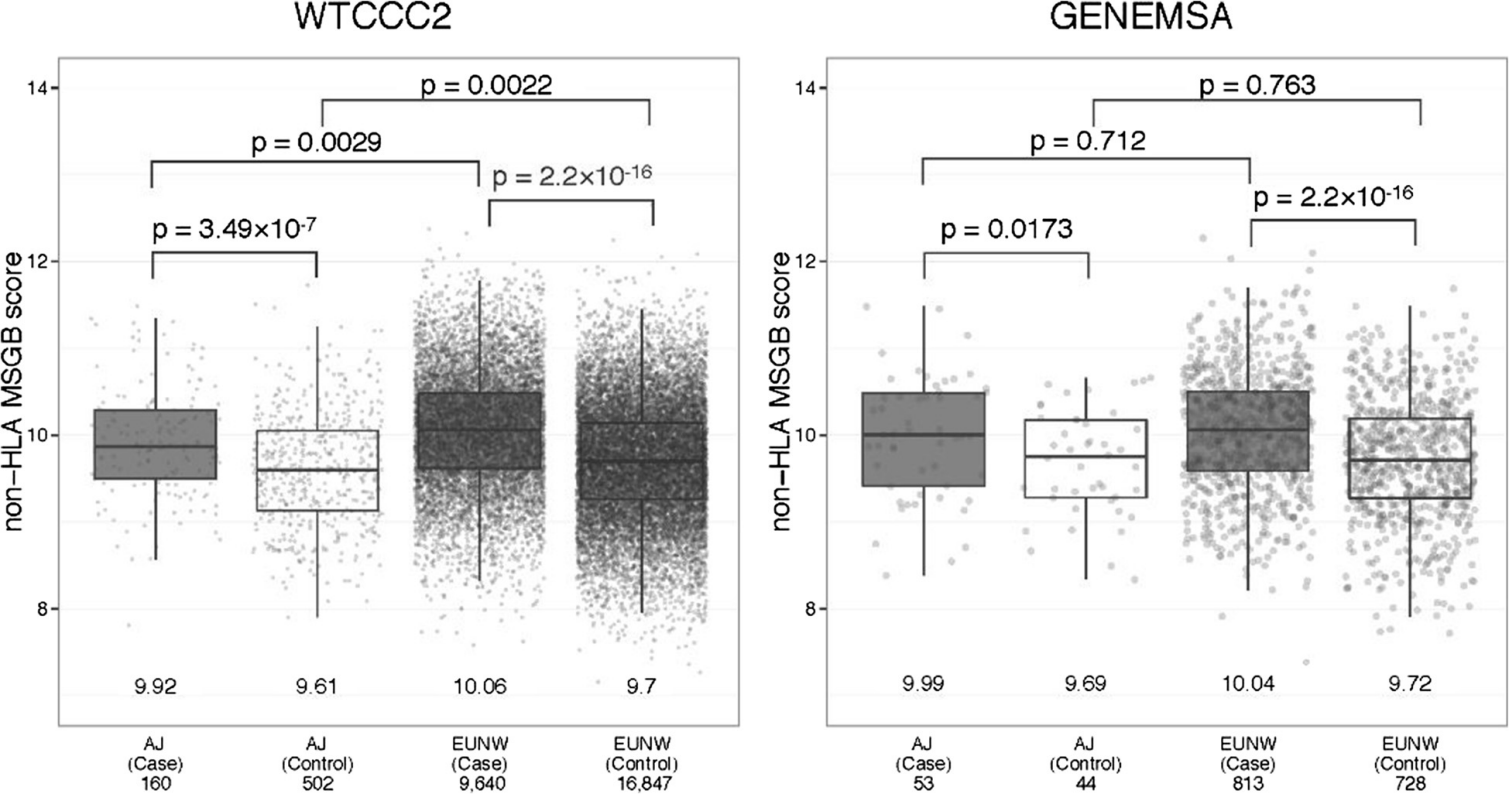
Multiple sclerosis genetic burden. Multiple sclerosis genetic burden in multiple sclerosis patients and controls of Ashkenazi Jewish (AJ) origin and Europeans (EUNW). The mean MSGB for each group is displayed above the graph. The number of samples in each group is displayed beside each box-plot

110 genome-wide SNPs and a polygenic risk score (MSGB) were used to assess genetic risk of MS. The mean MSGB was significantly higher in Ashkenazi cases than Ashkenazi controls (WTCCC2: difference d = 0.31, p = 3.5*10^−7^; GENEMSA d = 0.30, p = 0.017) (Fig. 6). As expected, a similar difference (d) was seen between EUNW cases and EUNW controls (WTCCC2: d = 0.37, p < 2.2*10^−16^; GENEMSA: d = 0.32, p < 2.2*10^−16^) (Fig. 6). Following the pattern observed for HLA risk, the non-HLA MSGB was lower in Ashkenazi cases than EUNW cases in both datasets (WTCCC2: d = 0.14, p = 0.0029; GENEMSA: d = 0.05, p = 0.7) but was statistically significant in only one of two datasets. The non-HLA MSGB was also lower in Ashkenazi controls than in the EUNW controls (WTCCC2: d = 0.08, p = 0.0022; GENEMSA: d = 0.03, p = 0.7) but was statistically significant in only one of the two datasets. Therefore, it is again not surprising that the percent of risk explained by the MSGB is lower in Ashkenazi (GENEMSA r^2^ = 0.024, WTCCC2 r^2^ = 0.011) than in non-Ashkenazi Europeans (GENEMSA r^2^ = 0.068, WTCCC2 r^2^ = 0.087).

Earlier, we have shown using a genome-wide identity-by-descent metric that within the country-based WTCCC2 dataset, the nearest European populations to the Ashkenazi Jews are Spanish and Italian cases and controls (Fig. 2), but the Ashkenazi cases had significantly lower non-HLA MSGB compared to the Italian and Spanish (cases (p = 0.002), whereas Italian and Spanish cases showed no significant difference from the EUNW cases. The Ashkenazi Jews controls had lower non-HLA MSGB than controls from Italy (p = 0.04). Controls from Italy showed no significant difference from the EUNW controls. These observations, altogether, suggest a larger missing heritability in Ashkenazi MS. To screen the genome for genetic susceptibility to MS in an all-Ashkenazi dataset, a genome-wide case–control association study of SNPs was performed on the GENEMSA (n = 53 cases, 42 controls) and WTCCC2 (n = 136 cases, 429 controls) datasets, and a genome-wide transmission disequilibrium test was performed on the IMSGC dataset (n = 76 trios). There were no significant results at a genome-wide FDR < 0.1 in any study and the meta-analysis of the three studies yielded no significant results at a genome-wide FDR < 0.1, which is not surprising given the limited power of this dataset.

## Discussion

A 2006 survey of MS frequency in Israel reported that the highest disease rates were in Israeli-born Jews and in Jewish immigrants from Europe/America, with prevalence similar to that seen in Europeans [5]. Jewish immigrants from African/Asian countries and Christian Arabs had intermediate MS rates (significantly lower than in the first two groups but not significantly different from each other) [5]. Moslem Arabs, Druze, and Bedouins had the lowest rates of MS. Karni and colleagues [15] highlight an intriguing difference in prevalence between Jewish immigrants from Africa/Asian countries (low prevalence) and their Israeli born children (higher prevalence) suggesting a strong environmental influence acting across a single generation [15, 16]. The HLA-DRB1*15:01 allele has been shown to be a risk allele in Ashkenazi [13], but it is less frequent in Ashkenazi (~5 %) [11, 16] compared to the general European population (~15-20 %), which is a counterintuitive observation given the high frequency of MS among Ashkenazi.

In this study, we have extracted Ashkenazi individuals using genetic data from three datasets. A key limitation of this study is the sample size, the small number of identified Ashkenazi individuals leaves little power for discovery of new variants, especially at the genome-wide level. Another limitation the small sample size of the well-characterized Ashkenazi controls [9] used to help define Ashkenazi from within our MS datasets. A larger set of well-characterized controls may allow the identification of more Ashkenazi individuals, and may make the identification more reproducible across future studies.

Despite the power limitation, we have confirmed that the classical MS determinant HLA-DRB1*15:01 is a risk allele in Ashkenazi. SNP-based *HLA* analysis using validated imputed techniques revealed the well-known HLA-DRB1*15:01- HLA-DQB1*06:02 association with risk was significant in Ashkenazi, but its frequency in Ashkenazi cases is significantly lower than the frequency in other European cases. The linked class I HLA-B*38:01 and HLA-C*12:03 alleles showed a nominally significant protective effect, which is in line with a previously described protective effect of HLA-B*38:01 in a very large European study [17]. Also relevant, a *HLA-B38* protective effect was detected in an Iranian MS population [18]. The HLA-A*68:02 allele showed a nominally significant risk effect. This allele belongs to multiple haplotypes [19] and is linked to previously described class II risk alleles, HLA-DRB1*13:01 [6, 13], HLA-DRB1*03:01 [6] and HLA-DRB1*15:03 in African Americans [20].

To our knowledge, this is the first study to validate HLA imputation performed on Ashkenazi samples using a European reference population to train the model. We have seen that generally imputation works well for most alleles of most HLA genes, but works very poorly for specific alleles (for example, DRB1*11:01, see Additional file 3 for further examples), which represents a limitation of this study. Other studies using HLA imputation to derive HLA alleles for Ashkenazi would likely benefit from masking those specific alleles, rather than trying to set a single threshold on the confidence metric of imputation. Ideally, an all-Ashkenazi reference population would be used to train the model.

The historical record shows that Jewish people emigrated in mass from their ancestral home in the Eastern Mediterranean, beginning over two and a half millennia ago, establishing Jewish communities in many different regions across the globe. The contemporaneous Jewish population is generally grouped according to the most recent place of origin into two main groups, Ashkenazi (originating from Eastern, Central, and Northern Europe), and Non-Ashkenazi (from North Africa, the Middle East and Asia). Members of each group differ in physiognomy and life style suggesting significant admixture as the force driving Jewish population diversity. However, recent genome-wide assessment of multiple Jewish datasets note their considerable degree of genetic homogeneity and closeness to other populations of the Levant, especially the Druze and Palestinians. Our own data using primarily UK and American Ashkenazi genomes suggests a degree of genome-wide similarity between Ashkenazi and Mediterranean in the context of a primarily northwest European cohort consistent with previous findings. Portions of genetic susceptibility to MS in Ashkenazi are shared with Europeans (HLA-DRB1*15:01 for example), while others are shared with another middle eastern population (HLA-B*38 and HLA-C*12 for example), consistent with their previously reported shared ancestry [9, 12].

'The non-HLA polygenic risk score conferring risk in Ashkenazi was lower in Ashkenazi cases than European cases, and, altogether, the previously described and validated risk alleles (DRB1*15:01 and genome-wide) explain a relatively smaller fraction of the genetic susceptibility to MS in Ashkenazi. Comparable differences were found between Ashkenazi controls and European controls, indicating that underlying differences in the healthy population can explain the apparent genomic differences in cases. The precise reason for the decreased concentration of MS susceptibility alleles in Ashkenazi Jews is unknown, and additional research is necessary to resolve the effects of selection and drift in the context of cultural isolation, admixture and migration. In this study, we also attempted to perform disease association analyses in a small Ashkenazi cohort. We found no evidence of SNP-level association using a genome-wide SNP-based analysis due most likely to lack of power, a rather important limitation of this study. Well-powered GWAS, re-sequencing and epidemiological studies in Ashkenazi datasets may provide a unique opportunity to further decipher the genetic and gene-environment underpinnings of MS.

## Methods

### Sources of data

The WTCCC2 dataset was provided by a collaboration between the International MS Genetics Consortium and the Wellcome Trust Case Control Consortium. This GWAS dataset consisted of 30,248 individuals [6]. This included 11,376 cases diagnosed with MS, as well as 18,872 controls, all of European ancestry. Cases were collected from reference centers in Australia, Belgium, Denmark, Finland, France, Germany, Ireland, Italy, New Zealand, Norway, Poland, Spain, Sweden, the UK, and the USA. Controls were collected from 12 centers. Genotyping was performed using Illumina Human 660-Quad chip in cases and Illumina Human 1.2 M - Duo chip in controls. Extensive quality control analyses were performed to reduce population stratification and inflation.

The GENEMSA dataset included 1,857 European ancestry individuals from a case–control GWAS [21] and consisted of 975 cases and 882, age- and gender-matched controls. Cases and controls were collected from reference centers in the USA, Netherlands, and Switzerland. Genotyping was performed with Illumina Human Hap 550 K BeadChip.

The IMSGC dataset was provided by the International MS Genetics Consortium. This trio dataset included 2,790 European ancestry individuals from a family-based genome-wide study and consisted of 930 MS cases and their unaffected parents [22]. Trios were collected at reference sites in the USA and the UK. Genotyping was performed using Affymetrix GeneChip Human Mapping 500 K arrays.

In all datasets, MS cases were diagnosed by neurologists specialized in demyelinating diseases in accordance with well-established diagnostic and study inclusion criteria [23]. Study protocols were approved by the local Committees on Human Research and Informed consent was obtained from all participants.

### Defining European Jewish ancestry

Europeans can be stratified into ancestral subgroups using a set of SNP markers from across the genome [8, 24–27]. In order to identify Ashkenazi individuals included within the three study datasets, genome-wide SNP data from a set of ethnically well-characterized Ashkenazi controls [9] were merged with the study datasets. After removing SNPs with minor allele frequency < 1 % and SNPs with > 0.1 % missing calls, 47,251 independent SNPs (with pair-wise r^2^ < 0.1) from the merged dataset were used to calculated identity by descent (IBD) using software Beagle [28], aggregated over ten rounds. Multidimensional scaling [29] (MDS) with 50 dimensions and hierarchical Ward [30] clustering using IBD distances was performed using R software. Definition of Ashkenazi was performed separately in each of the three datasets (GENEMSA, WTCCC2, IMSGC). Ashkenazi ancestry was defined using hierarchical clustering in the case–control datasets (GENEMSA and WTCCC2) and using MDS in the trio study (IMSGC) due to the inherent interrelatedness of individuals in the trio design.

### HLA imputation

In each of the three datasets, SNP information was used to impute classical HLA alleles at 5 loci: *A*, *B*, *C*, *DRB1*, and *DQB1*. The open-source R package HiBAG software was used for imputation. Briefly, an ensemble classifier is created consisting of individual classifiers with HLA and SNP haplotype probabilities estimated from bootstrapped samples and SNP subsets and HLA type predictions are averaged over the posterior probabilities from all classifiers. The published model for populations of European ancestry was used. Validation of HLA imputation was performed on a subset of 806 Europeans and 94 Ashkenazi from the GENEMSA dataset using sequence-based genotyping. Due to differences in LD decay and allele frequency between Ashkenazi and Europeans, quality control was performed at each allele of each locus in order to minimize the error rate while maximizing the call rate (see Additional file 3).

### Polygenic risk score

As of July 2014, SNPs at 110 non-HLA variants had confirmed associations with MS [7, 31] (Additional file 1: Table S1), each with a much weaker conferred risk (odds ratio ≈ 1.2-1.4) than HLA-DRB1*1501. For each individual, a multiple sclerosis genetic burden, MSGB [31], was calculated across these non-HLA genes. Briefly, the MSGB is a polygenic risk score, the weighted sum of the number of risk alleles carried, where weights are determined by log of published odds ratios. Differences of MSGB score between cases and controls were tested by one-sided Wilcoxon test because the MSGB score consists of risk alleles for MS.

### Disease association studies in Ashkenazi Jews

To look for MS genetic susceptibility loci that are specific to Ashkenazi Jews, Ashkenazi Jewish individuals from each of the three datasets were extracted. A meta-analysis genome-wide study on all SNPs and a meta-analysis of imputed HLA alleles was then performed. For SNPs in the WTCCC2 study and the GENEMSA study, genome-wide logistic regression was performed with age, gender, and PCA covariates. For HLA alleles in the GENEMSA and WTCCC studies, genotypes of each allele (e.g. HLA-DRB1*15:01) were re-coded as 0, 1, or 2 copies and logistic regression was performed. For SNPs in the IMSGC trio study, a transmission disequilibrium (TD) test was performed. For HLA alleles in the IMSGC study, genotypes were re-coded as 0, 1, or 2 copies and at TD test was performed. Logistic regression, TD tests, and random-effects meta-analysis were implemented in Plink.

## Additional files

Additional file 1:
**Table S1.** The 110 previously identified variants associated with MS, and the nearest genes. (DOC 166 kb) Additional file 2:
**Clinical Characteristics of Ashkenazi.** (DOCX 39 kb) Additional file 3:
**HLA imputation.** (DOC 819 kb) Additional file 4:
**Figure S1.** Principal component analysis of the IMSGC trio dataset using genome-wide IBD distances, showing component 1 (x-axis) versus component 2 (y-axis). The first principal component (PC1) differentiates Ashkenazi (higher values) from other Europeans (lower values). Probands are highlighted as shaded red circles (females) or squared (males). Unaffected parents are highlighted as open red circles (mothers) and squares (fathers). Parents are connected to probands by dashed lines. In each successive picture a different set of trios is highlighted (since highlighting all trios at once makes the figureunreadable). Panels 2 thru 6 demonstrate probands of partial Jewish Ancestry (intermediate values of the PC-1 axis) may have a single Jewish parent and a single non-Jewish parent, or they may have two parents each of partial Jewish ancestry. (XLSX 191 kb) Additional file 5:
**Supplementary figure PCA of IMSGC.** (PDF 230 kb)
