# Supplementary material for: Genetic contribution to multiple sclerosis risk among Ashkenazi Jews
Source: BMC Med Genet. 2015 Jul 28;16:55. doi: 10.1186/s12881-015-0201-2 (PMC4557862; doi:10.1186/s12881-015-0201-2)
Supplement: Additional file 1: — Table S1. The 110 previously identified variants associated with MS, and the nearest genes. (DOC 166 kb) [file 12881_2015_201_MOESM1_ESM.doc]

## Table S1 - The 110 previously identified variants associated with MS, and the nearest genes.

| **SNP ID** | Chr | Position (hg18) | Risk Allele | Effect size | Nearest Genes (distance) |
| --- | --- | --- | --- | --- | --- |
| **rs3748817** | 1 | 2515525 | A | 1.14 | MMEL1 |
| **rs3007421** | 1 | 6452776 | A | 1.12 | PLEKHG5 |
| **rs12087340** | 1 | 85519581 | A | 1.22 | LOC646626 (3222), DDAH1 (37175) |
| **rs11587876** | 1 | 85687771 | A | 1.12 | DDAH1 |
| **rs41286801** | 1 | 92748052 | A | 1.2 | EVI5 |
| **rs7552544** | 1 | 101013481 | A | 1.08 | VCAM1 (36292), EXTL2 (97035) |
| **rs11581062** | 1 | 101180107 | G | 1.05 | VCAM1 |
| **rs6677309** | 1 | 116881689 | A | 1.34 | CD58 |
| **rs666930** | 1 | 120060493 | G | 1.09 | PHGDH |
| **rs2050568** | 1 | 156036865 | G | 1.08 | FCRL1 |
| **rs35967351** | 1 | 158978428 | A | 1.09 | SLAMF7 |
| **rs1359062** | 1 | 190808095 | C | 1.18 | RGS21 (205058), RGS1 (3385) |
| **rs55838263** | 1 | 199141351 | A | 1.12 | C1orf106 |
| **rs4665719** | 2 | 24871364 | G | 1.09 | CENPO |
| **rs2163226** | 2 | 43214760 | A | 1.1 | HAAO (341505), ZFP36L2 (88285) |
| **rs842639** | 2 | 60948749 | A | 1.11 | FLJ16341 |
| **rs7595717** | 2 | 68440981 | A | 1.1 | CNRIP1 (40294), PLEK (4845) |
| **rs17174870** | 2 | 112381672 | G | 1.03 | MERTK |
| **rs9967792** | 2 | 191682680 | G | 1.11 | STAT4 |
| **rs9989735** | 2 | 230823698 | C | 1.17 | SP140 |
| **rs11719975** | 3 | 18760589 | C | 1.09 | SATB1 (305320), KCNH8 (404432) |
| **rs2371108** | 3 | 27732022 | A | 1.08 | EOMES |
| **rs1813375** | 3 | 28053575 | A | 1.15 | EOMES (314786), CMC1 (204553) |
| **rs4679081** | 3 | 32988487 | G | 1.08 | CCR4 (17080), GLB1 (24617) |
| **rs9828629** | 3 | 71613036 | G | 1.08 | FOXP1 |
| **rs2028597** | 3 | 107041527 | G | 1.04 | CBLB |
| **rs1131265** | 3 | 120705146 | C | 1.19 | TIMMDC1 |
| **rs1920296** | 3 | 123026267 | C | 1.14 | IQCB1 |
| **rs2255214** | 3 | 123253229 | C | 1.11 | ILDR1 (29412), CD86 (3670) |
| **rs9282641** | 3 | 123279458 | G | 1.12 | CD86 |
| **rs1014486** | 3 | 161173806 | G | 1.11 | IQCJ-SCHIP1 (75957), IL12A (15511) |
| **rs7665090** | 4 | 103770651 | G | 1.08 | NFKB1 (13144), MANBA (1040) |
| **rs2726518** | 4 | 106392648 | C | 1.09 | TET2 |
| **rs6881706** | 5 | 35914913 | C | 1.12 | IL7R (2233), CAPSL (25242) |
| **rs6880778** | 5 | 40434853 | G | 1.1 | DAB2 (973761), PTGER4 (280936) |
| **rs71624119** | 5 | 55476487 | G | 1.12 | ANKRD55 |
| **rs756699** | 5 | 133474474 | A | 1.12 | VDAC1 (105751), TCF7 (3827) |
| **imm_5_141486748** | 5 | 141486748 | C | 1.07 | NDFIP1 |
| **rs2546890** | 5 | 158692478 | A | 1.06 | LOC285626 |
| **rs4976646** | 5 | 176721176 | G | 1.13 | RGS14 |
| **rs17119** | 6 | 14827475 | A | 1.11 | CD83 (582348), JARID2 (526710) |
| **rs941816** | 6 | 36483282 | G | 1.13 | PXT1 |
| **rs72928038** | 6 | 91033489 | A | 1.11 | BACH2 |
| **rs802734** | 6 | 128320491 | A | 1.03 | THEMIS (39022), PTPRK (11126) |
| **rs11154801** | 6 | 135781048 | A | 1.11 | AHI1 |
| **rs17066096** | 6 | 137494601 | G | 1.14 | IL20RA (86610), IL22RA2 (12049) |
| **rs7769192** | 6 | 138004348 | G | 1.08 | OLIG3 (147124), LOC100130476 (182152) |
| **rs67297943** | 6 | 138286509 | A | 1.12 | TNFAIP3 (40367), PERP (164826) |
| **rs212405** | 6 | 159390547 | T | 1.15 | TAGAP (4375), FNDC1 (119870) |
| **rs1843938** | 7 | 3079560 | A | 1.08 | CARD11 (29525), SDK1 (228046) |
| **rs706015** | 7 | 26981513 | C | 1.14 | SKAP2 (110647), HOXA1 (117626) |
| **rs917116** | 7 | 28139264 | C | 1.12 | JAZF1 |
| **rs60600003** | 7 | 37348990 | C | 1.16 | ELMO1 |
| **rs201847125** | 7 | 50296113 | G | 1.11 | C7orf72 (126715), IKZF1 (18811) |
| **rs354033** | 7 | 148920397 | G | 1.03 | ZNF767 |
| **rs1021156** | 8 | 79738359 | A | 1.12 | PKIA (58302), ZC2HC1A (2478) |
| **rs2456449** | 8 | 128262163 | G | 1.1 | PCAT1 (159722), POU5F1B (234876) |
| **rs4410871** | 8 | 128884211 | G | 1.12 | PVT1 |
| **rs759648** | 8 | 129228127 | C | 1.09 | PVT1 (45446), MIR1208 (3417) |
| **rs2150702** | 9 | 5883861 | G | 1.16 | MLANA |
| **rs2104286** | 10 | 6139051 | A | 1.21 | IL2RA |
| **rs793108** | 10 | 31455112 | A | 1.09 | ZNF438 (94240), ZEB1-AS1 (190351) |
| **rs2688608** | 10 | 75328355 | A | 1.07 | CAMK2G (24000), C10orf55 (11378) |
| **rs1782645** | 10 | 80718617 | A | 1.09 | ZMIZ1 |
| **rs7923837** | 10 | 94471897 | G | 1.11 | HHEX (26509), EXOC6 (112553) |
| **rs7120737** | 11 | 47658971 | G | 1.13 | AGBL2 |
| **rs34383631** | 11 | 60549906 | A | 1.11 | CD6 (5482), CD5 (76600) |
| **rs694739** | 11 | 63853809 | A | 1.08 | PRDX5 (7938), CCDC88B (10457) |
| **rs533646** | 11 | 118071956 | G | 1.1 | TREH (16365), DDX6 (51727) |
| **rs9736016** | 11 | 118230104 | T | 1.1 | DDX6 (62922), CXCR5 (29581) |
| **rs523604** | 11 | 118260948 | A | 1.09 | CXCR5 |
| **rs1800693** | 12 | 6310270 | G | 1.14 | TNFRSF1A |
| **rs12296430** | 12 | 6373761 | C | 1.14 | LTBR (2768), CD27-AS1 (44667) |
| **rs11052877** | 12 | 9796957 | G | 1.1 | CD69 |
| **rs201202118** | 12 | 56468329 | A | 1.14 | TSFM |
| **rs7132277** | 12 | 122159335 | A | 1.1 | PITPNM2 |
| **rs4772201** | 13 | 98884260 | A | 1.12 | MIR548AN (27705), TM9SF2 (67469) |
| **rs2236262** | 14 | 68331225 | A | 1.08 | ZFP36L1 |
| **rs4903324** | 14 | 75031264 | A | 1.1 | JDP2 (22107), BATF (27273) |
| **rs74796499** | 14 | 87502081 | C | 1.31 | GALC |
| **rs12148050** | 14 | 102333541 | A | 1.08 | TRAF3 |
| **rs59772922** | 15 | 76994521 | A | 1.11 | MORF4L1 (17385), CTSH (6626) |
| **rs8042861** | 15 | 88778337 | A | 1.08 | IQGAP1 |
| **rs2744148** | 16 | 1013553 | G | 1.09 | SOX8 (36573), SSTR5-AS1 (40530) |
| **rs12927355** | 16 | 11102272 | G | 1.21 | CLEC16A |
| **rs4780346** | 16 | 11196307 | A | 1.09 | CLEC16A (12760), SOCS1 (59468) |
| **rs6498184** | 16 | 11343491 | G | 1.15 | PRM1 (60798), RMI2 (3321) |
| **rs7204270** | 16 | 30064464 | G | 1.09 | MAPK3 (22333), CORO1A (37768) |
| **rs1886700** | 16 | 67243406 | A | 1.11 | CDH3 |
| **rs12149527** | 16 | 77668097 | A | 1.08 | WWOX |
| **rs7196953** | 16 | 78206895 | A | 1.08 | MAF (14772), DYNLRB2 (925460) |
| **rs35929052** | 16 | 84551985 | G | 1.14 | IRF8 (38273), LOC146513 (325553) |
| **rs12946510** | 17 | 35165903 | A | 1.08 | GRB7 (8839), IKZF3 (1591) |
| **rs4796791** | 17 | 37784289 | A | 1.1 | STAT3 |
| **rs4794058** | 17 | 42952097 | A | 1.07 | MRPL45P2 (27112), NPEPPS (11346) |
| **rs8070345** | 17 | 55171539 | A | 1.14 | VMP1 |
| **rs7238078** | 18 | 54535172 | A | 1.05 | MALT1 |
| **rs1077667** | 19 | 6619972 | G | 1.16 | TNFSF14 |
| **rs34536443** | 19 | 10324118 | C | 1.28 | TYK2 |
| **rs2288904** | 19 | 10603170 | G | 1.14 | SLC44A2 |
| **rs1870071** | 19 | 16366106 | G | 1.12 | EPS15L1 |
| **rs11554159** | 19 | 18146944 | G | 1.15 | IFI30 |
| **rs8107548** | 19 | 54562455 | G | 1.09 | DKKL1 |
| **rs4810485** | 20 | 44181354 | A | 1.08 | CD40 |
| **rs17785991** | 20 | 47872168 | A | 1.09 | SLC9A8 |
| **rs2248359** | 20 | 52224925 | G | 1.07 | CYP24A1 (1002), PFDN4 (32984) |
| **rs2256814** | 20 | 61844427 | A | 1.11 | SLC2A4RG |
| **rs6062314** | 20 | 61880157 | A | 1.1 | ZBTB46 |
| **rs2283792** | 22 | 20461125 | C | 1.08 | MAPK1 |
| **rs470119** | 22 | 49313780 | A | 1.07 | TYMP |
