# Supplementary material for: Genetic contribution to multiple sclerosis risk among Ashkenazi Jews
Source: BMC Med Genet. 2015 Jul 28;16:55. doi: 10.1186/s12881-015-0201-2 (PMC4557862; doi:10.1186/s12881-015-0201-2)
Supplement: Additional file 2: — Clinical Characteristics of Ashkenazi. (DOCX 39 kb) [file 12881_2015_201_MOESM2_ESM.docx]

## Clinical Characteristics of Ashkenazi

A detailed clinical database was available for the UCSF subset of cases from the GENEMSA dataset (54 AJ and 407 EUNW), and the UCSF subset of cases from the WTCCC dataset (125 AJ and 568 EUNW). The clinical characteristics of multiple sclerosis in AJ versus EUNW are summarized in the table. The multiple sclerosis severity score (MSSS) was significantly lower in AJ than in EUNW (Wilcoxon test: p = 0.0012, effect size r = 0.45). Compared to EUNW, a lower proportion of AJ reported motor weakness (64% vs. 74%, p = 0.042, Fisher's exact test, no correction for multiple comparisons) and acute transverse myelitis (12% vs. 23% p = 0.016).

AJ = European Jews, EUNW = northern and western Europeans.
