# Supplementary material for: Genetic contribution to multiple sclerosis risk among Ashkenazi Jews
Source: BMC Med Genet. 2015 Jul 28;16:55. doi: 10.1186/s12881-015-0201-2 (PMC4557862; doi:10.1186/s12881-015-0201-2)

## HLA imputation

The open-source R package HiBAG software was used for imputation. The published model for populations of European ancestry was used. This could potentially pose a problem because of the population mismatch between the samples to be imputed (Ashkenazi) and the reference (European). Furthermore, a large number of SNPs (~4500) were available for imputation in the GENEMSA dataset. However, a limited number of SNPs (~1000) was available in all three datasets (GENEMSA, WTCCC2, and IMSGC), and these were the SNPs used for imputation for the purpose of association analysis. The table below shows that imputation accuracy was somewhat lower for Ashkenazi samples than for the European samples, and that use of fewer SNPs decreased imputation for the *DRB1* gene but did not have a dramatic effect on the other genes.

| Imputation Accuracy | HLA-A | HLA-B | HLA-C | HLA-DRB1 | HLA-DQB1 |
| --- | --- | --- | --- | --- | --- |
| Europeans (all SNPs) | 98.1 | 96.7 | 98.9 | 93.2 | 90.1 |
| Europeans (limited SNPs) | 98.1 | 95.9 | 98.8 | 90.9 | 89.2 |
| Ashkenazi Jews (all SNPs) | 98.4 | 93.7 | 99.0 | 89.0 | 86.3 |
| Ashkenazi Jews (limited SNPs) | **97.4** | **92.6** | **98.4** | **83.2** | **84.7** |

The HiBAG program provides a quality metric for each allele imputed for each individual. For certain alleles, setting a higher threshold for the quality metric leads to better imputation accuracy. For other alleles, the quality metric is a poor indicator of imputation accuracy. Thus, we set a different threshold for the quality metric for each allele, as described below. The accuracy numbers reported in the table above are measured before the allele-specific quality controls described below.

For the *HLA-A* gene, the plot below shows the accuracy of imputation (y-axis) versus the call rate (x-axis) for different threshold of the confidence metric (color scale). A higher threshold value of the confidence metric will yield higher accuracy but lower call rate. In the case of *HLA-A*, we were able to achieve 97.5% accuracy and 100% call rate with the lowest threshold of the confidence metric. In order to achieve 100% accuracy, we would sacrifice 50% call rate, and thus the decision was made to set the lowest possible threshold of the confidence metric.


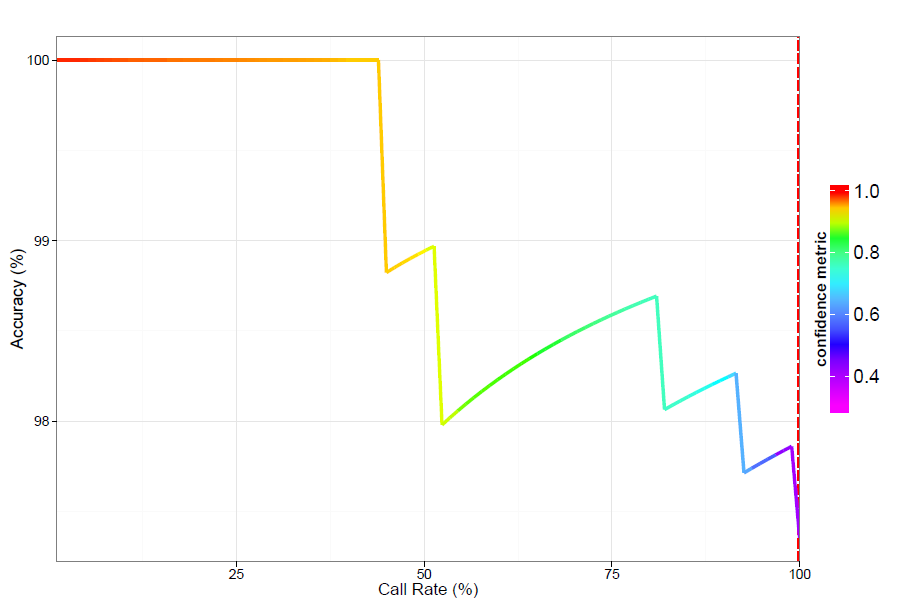


For the *HLA-B* gene, we chose a threshold of confidence metric that yielded 94% accuracy at 95% call rate. The threshold is delineated by the dashed red line.


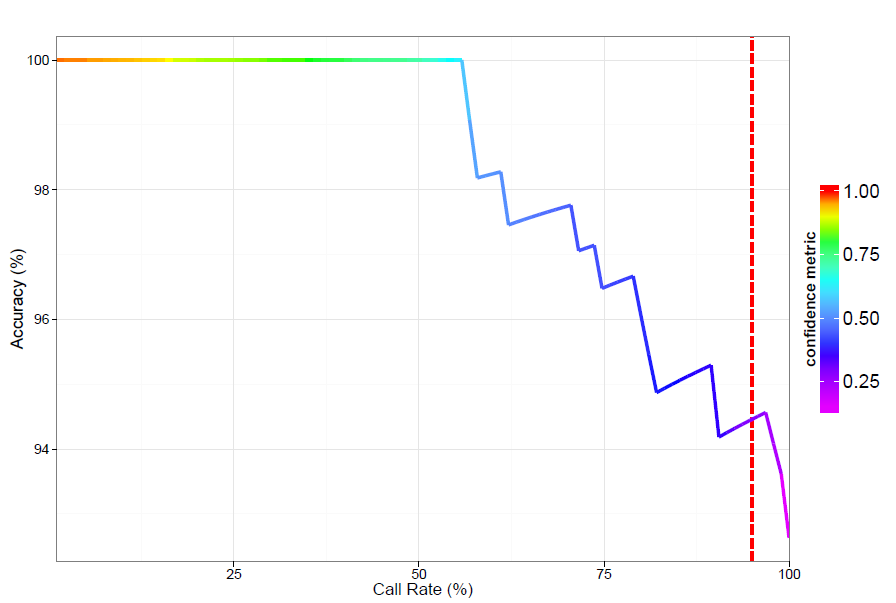


For *HLA-C*, there was a steep drop-off in accuracy at a threshold confidence metric of about 0.5. Thus the threshold was chosen to maintain 100% accuracy while achieving over 95% call rate.


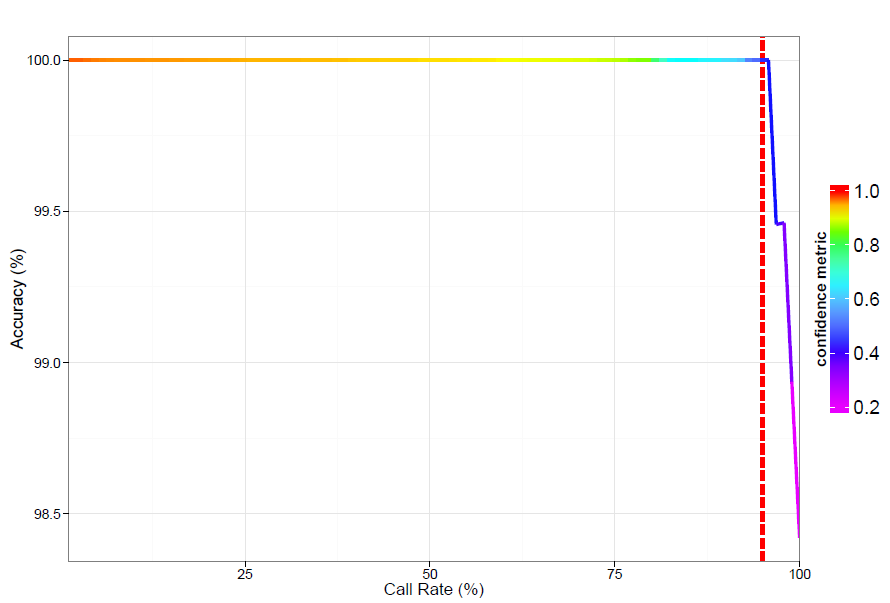


For *HLA-DRB1*, we were able to achieve 90% accuracy with a call rate of 80%. As shown in the plot below, higher levels of accuracy, say 95%, could not be achieved without a significance sacrifice of call rate to below 60%.


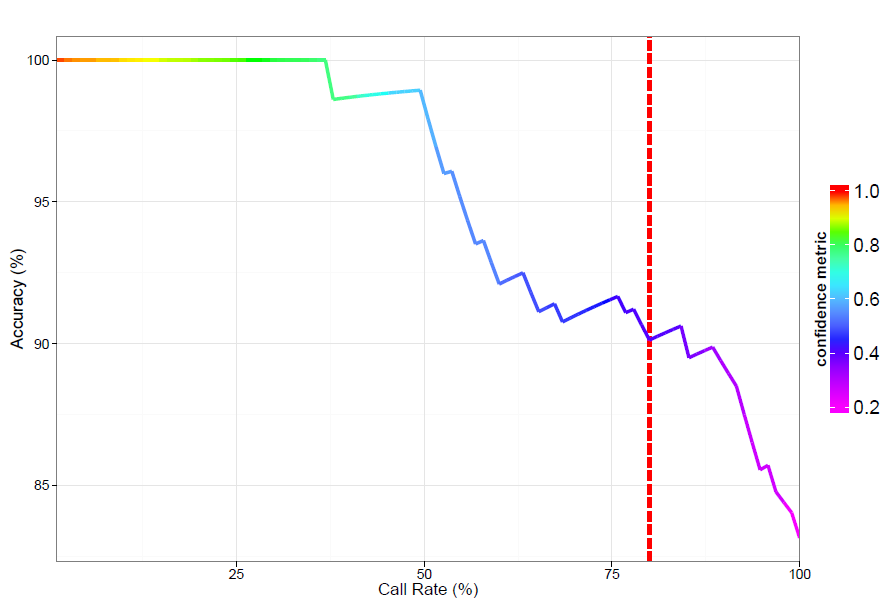


For *HLA-DQB1*, the confidence metric is a poor indicator of imputation accuracy. Thus, the lowest possible threshold was set on the confidence metric, achieving an accuracy of 85% with a call rate of 100%.


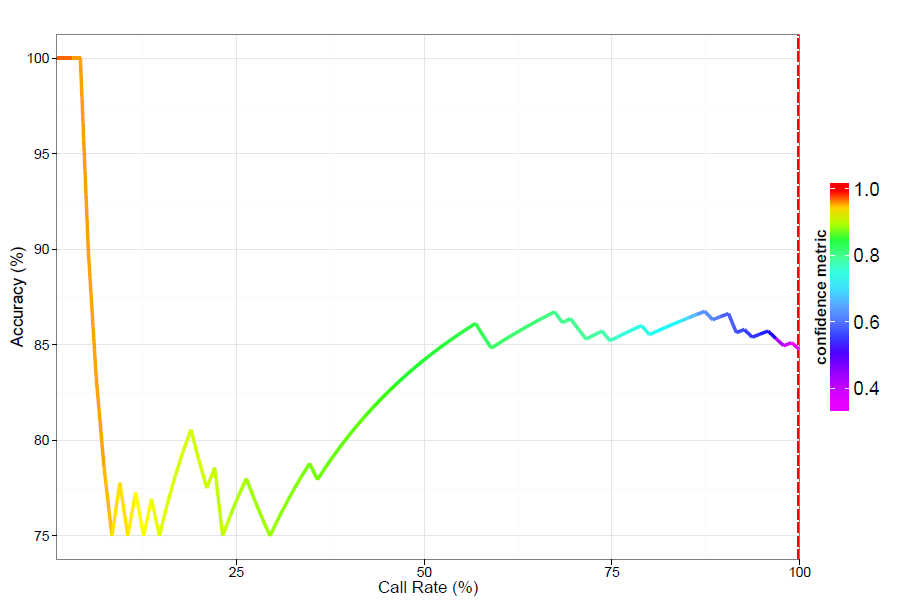


Due to inherent differences between allele frequencies in the Ashkenazi and European populations, certain alleles of certain genes are over-called by imputation in Ashkenazi. These alleles were removed before association analysis was performed. For *HLA-A* plot below, the height of the colored bars on the left represents the number of times each allele was predicted by imputation in the Ashkenazi (left) and European (right) samples. The black lines indicate the relative allele frequencies in the reference population used for imputation. This illustrates the dramatic difference in allele frequencies between Ashkenazi and Europeans.


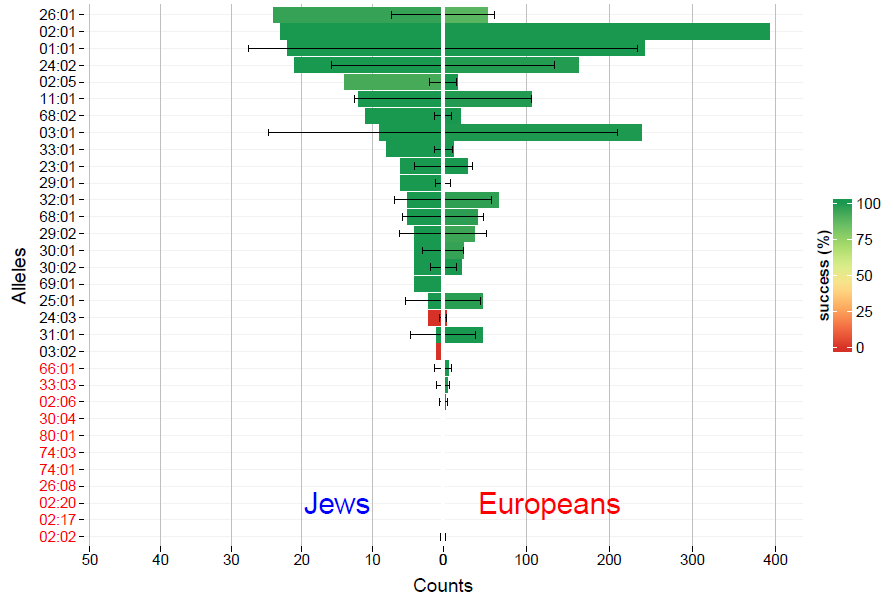


For *HLA-A*, the plot below shows the frequency of each allele in Ashkenazi as determined by genotyping (left) and imputation (right). The relative frequencies of most alleles are well preserved. However, certain alleles are under-called by imputation such as 24:03 and 03:02, while other alleles are over-called such as 02:02 and 25:01. These over-called alleles were not carried forward for association analysis.


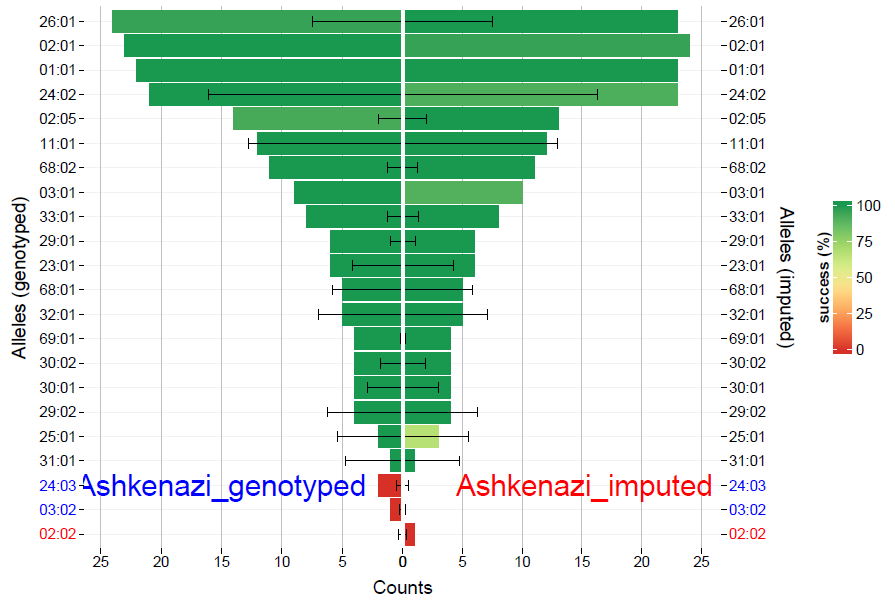


For *HLA-B* plot below, the height of the colored bars on the left represents the number of times each allele was predicted by imputation in the Ashkenazi (left) and European (right) samples, illustrating the dramatic difference in allele frequencies between Ashkenazi and Europeans.


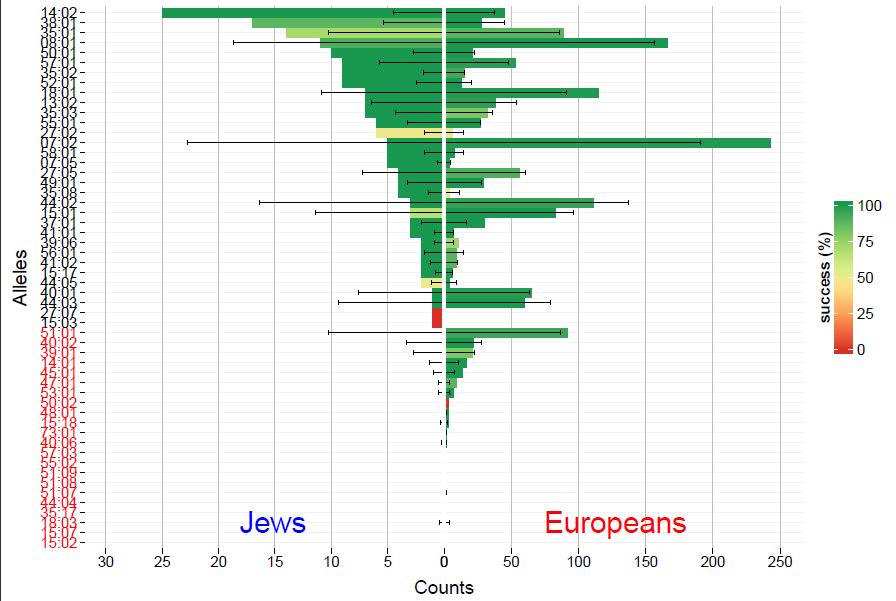


For *HLA-B*, the plot below shows the frequency of each allele in Ashkenazi as determined by genotyping (left) and imputation (right). The relative frequencies of most alleles are well preserved. However, certain alleles are under-called by imputation such as 27:02, 44:05, 27:07, and 15:03 and, while other alleles are over-called such as 35:08, 39:01, 40:02, and 15:18. The over-called alleles were not carried forward for association analysis.


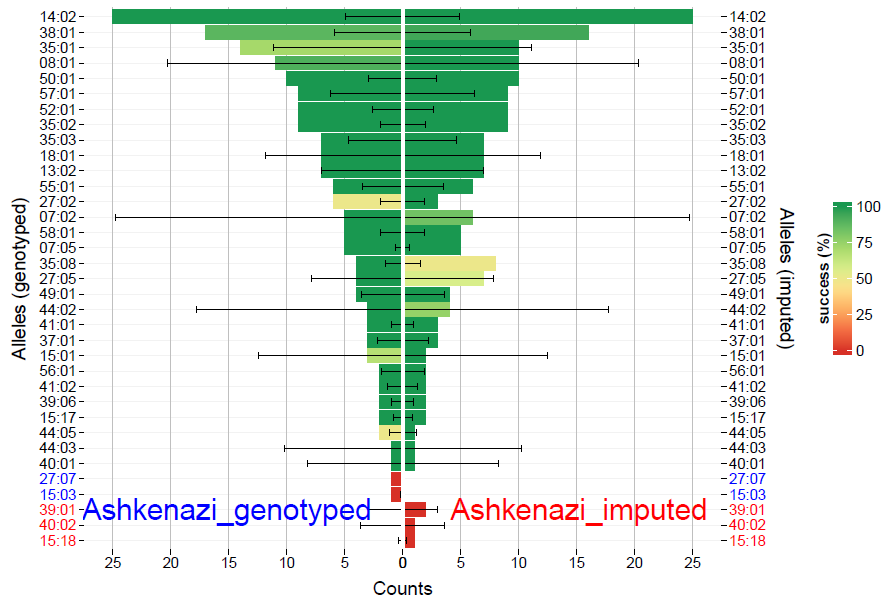


For *HLA-C* plot below, the height of the colored bars on the left represents the number of times each allele was predicted by imputation in the Ashkenazi (left) and European (right) samples, illustrating the dramatic difference in allele frequencies between Ashkenazi and Europeans.


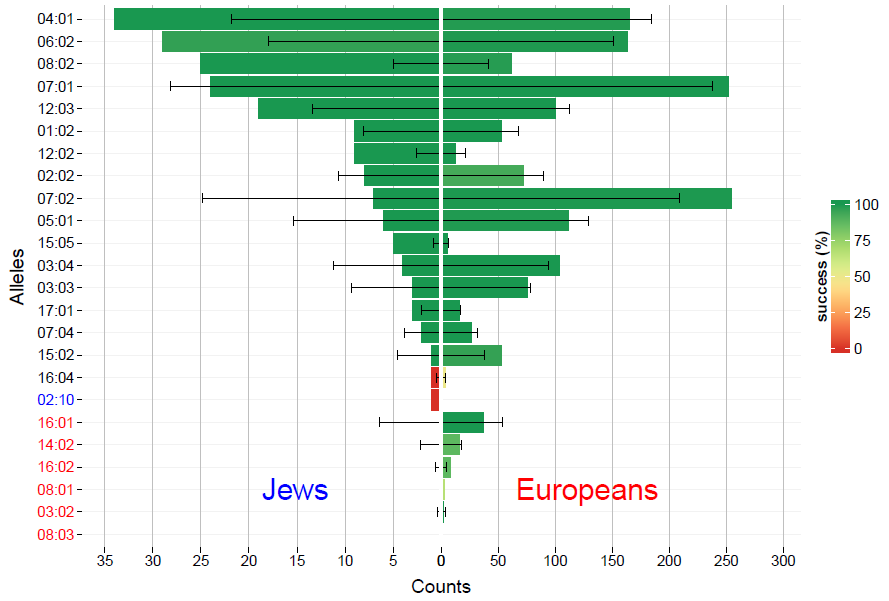


For *HLA-C*, the plot below shows the frequency of each allele in Ashkenazi as determined by genotyping (left) and imputation (right). The relative frequencies of most alleles are well preserved. However, certain alleles are under-called by imputation such as 16:04 and 02:10. while other alleles are over-called such as 03:04. The over-called alleles were not carried forward for association analysis.


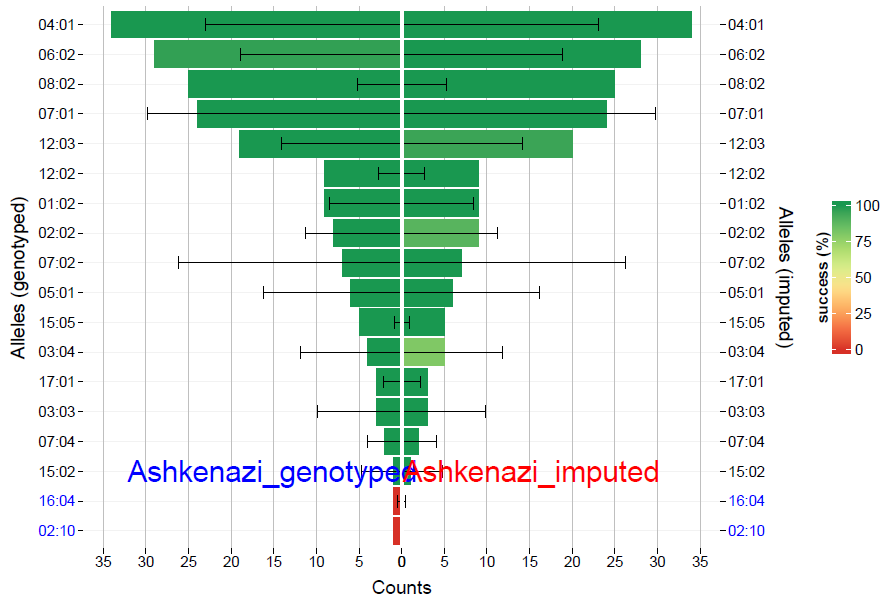


For *HLA-DRB1* plot below, the height of the colored bars on the left represents the number of times each allele was predicted by imputation in the Ashkenazi (left) and European (right) samples, illustrating the most dramatic difference in allele frequencies between Ashkenazi and Europeans.


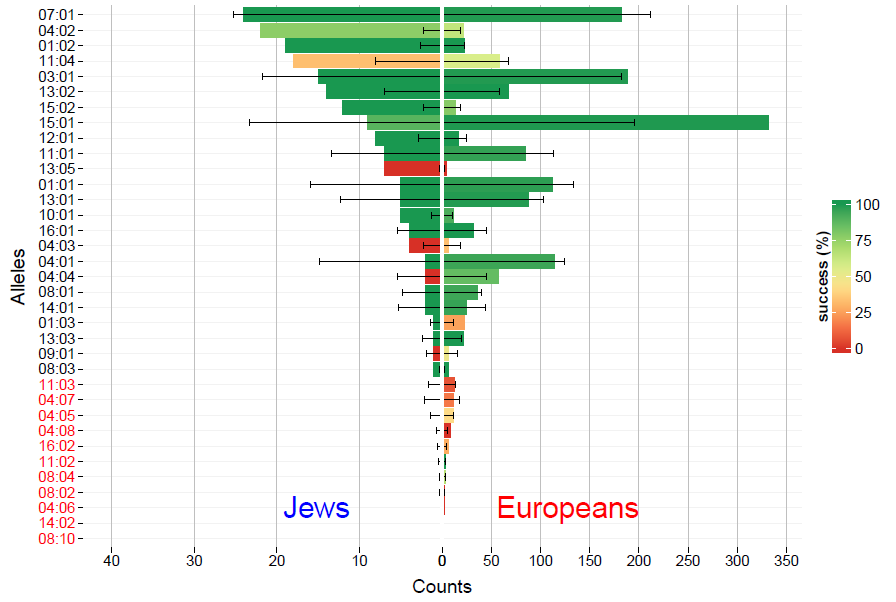


For *HLA-DRB1*, the plot below shows the frequency of each allele in Ashkenazi as determined by genotyping (left) and imputation (right). The relative frequencies of most alleles are well preserved. However, certain alleles are under-called by imputation such as 11:04, 13:05, 04:03, and 09:01, while other alleles are over-called such as 11:01 and 04:01. The over-called alleles were not carried forward for association analysis. Furthermore, the 04:04 allele was often misimputed (though not necessarily over-called or under-called) and was also not carried forward for association analysis.
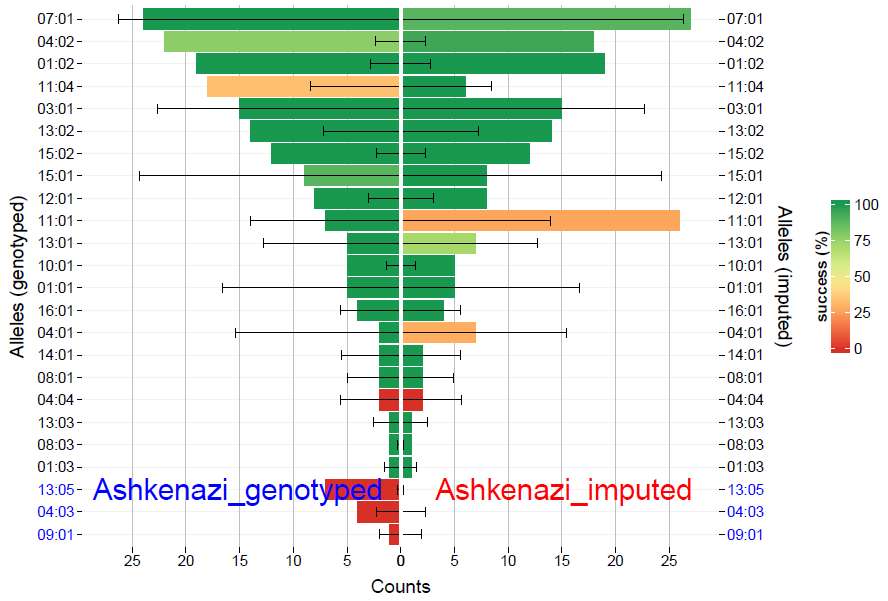


For *HLA-DQB1* plot below, the height of the colored bars on the left represents the number of times each allele was predicted by imputation in the Ashkenazi (left) and European (right) samples, illustrating the dramatic difference in allele frequencies between Ashkenazi and Europeans.


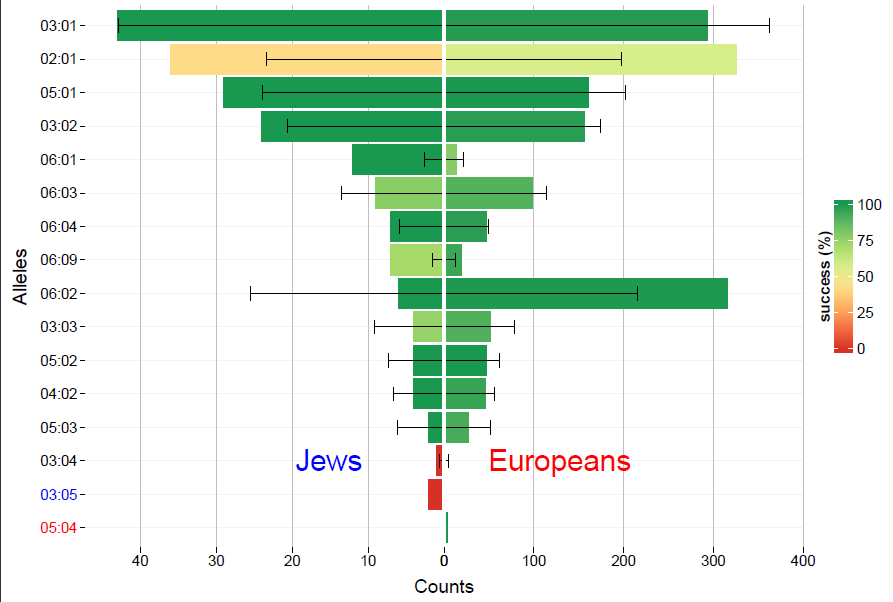


For *HLA-DQB1*, the plot below shows the frequency of each allele in Ashkenazi as determined by genotyping (left) and imputation (right). The relative frequencies of most alleles are well preserved. However, certain alleles are under-called by imputation such as 02:01, 03:05, and 03:04 while other alleles are over-called such as 02:02. The over-called allele was not carried forward for association analysis.


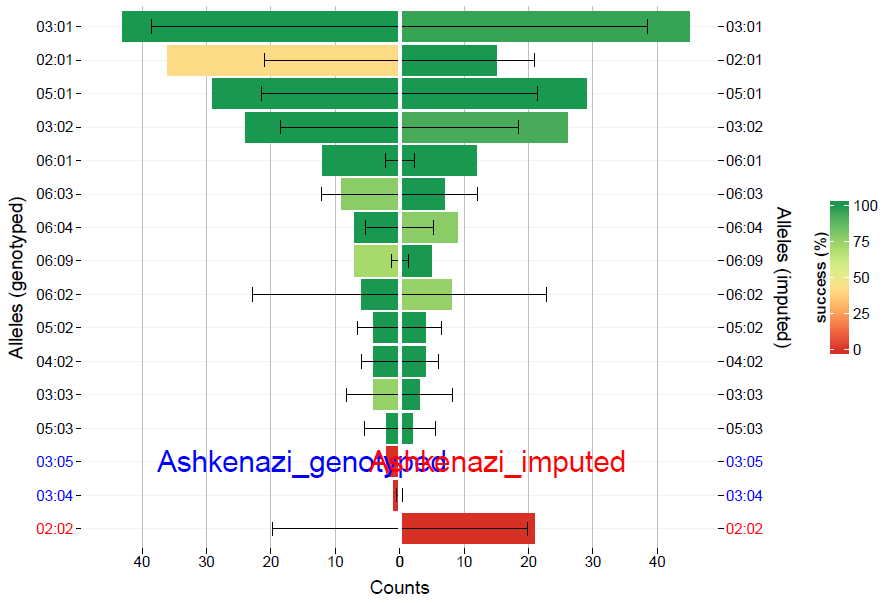

Supplement: Additional file 3: — HLA imputation. (DOC 819 kb) [file 12881_2015_201_MOESM3_ESM.doc]
