# Supplementary figures and images for: Genetic contribution to multiple sclerosis risk among Ashkenazi Jews
Source: BMC Med Genet. 2015 Jul 28;16:55. doi: 10.1186/s12881-015-0201-2 (PMC4557862; doi:10.1186/s12881-015-0201-2)

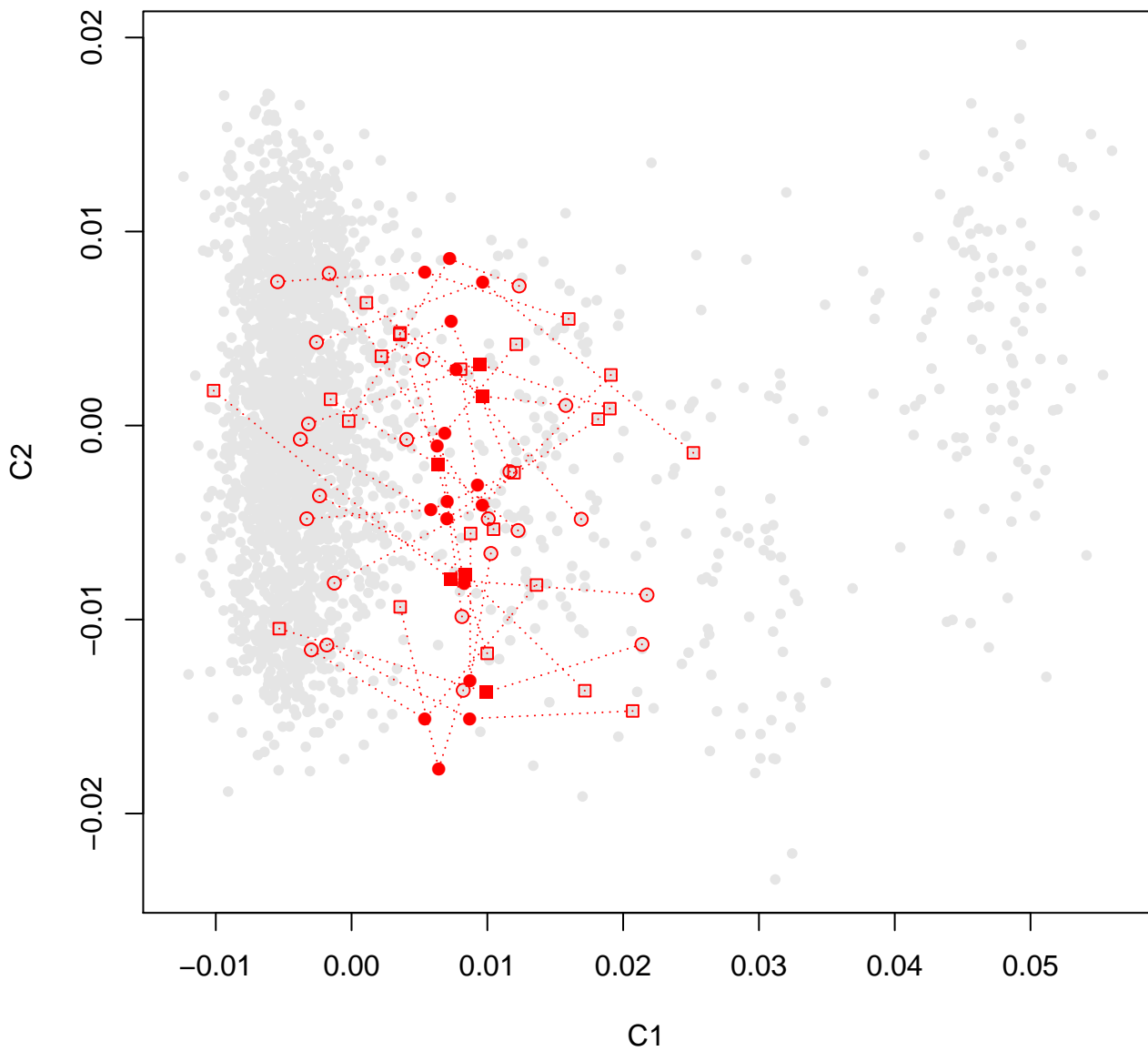

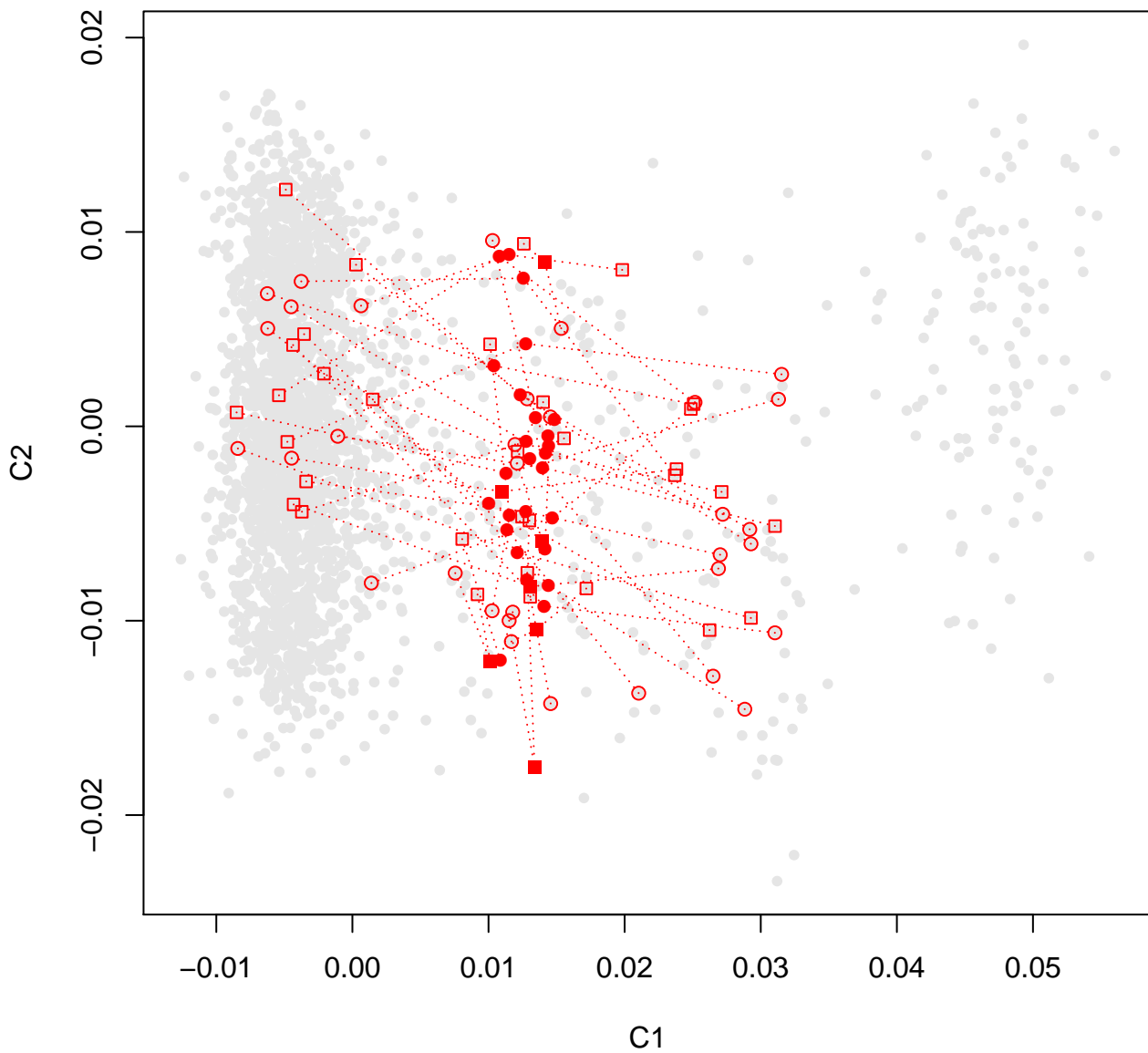

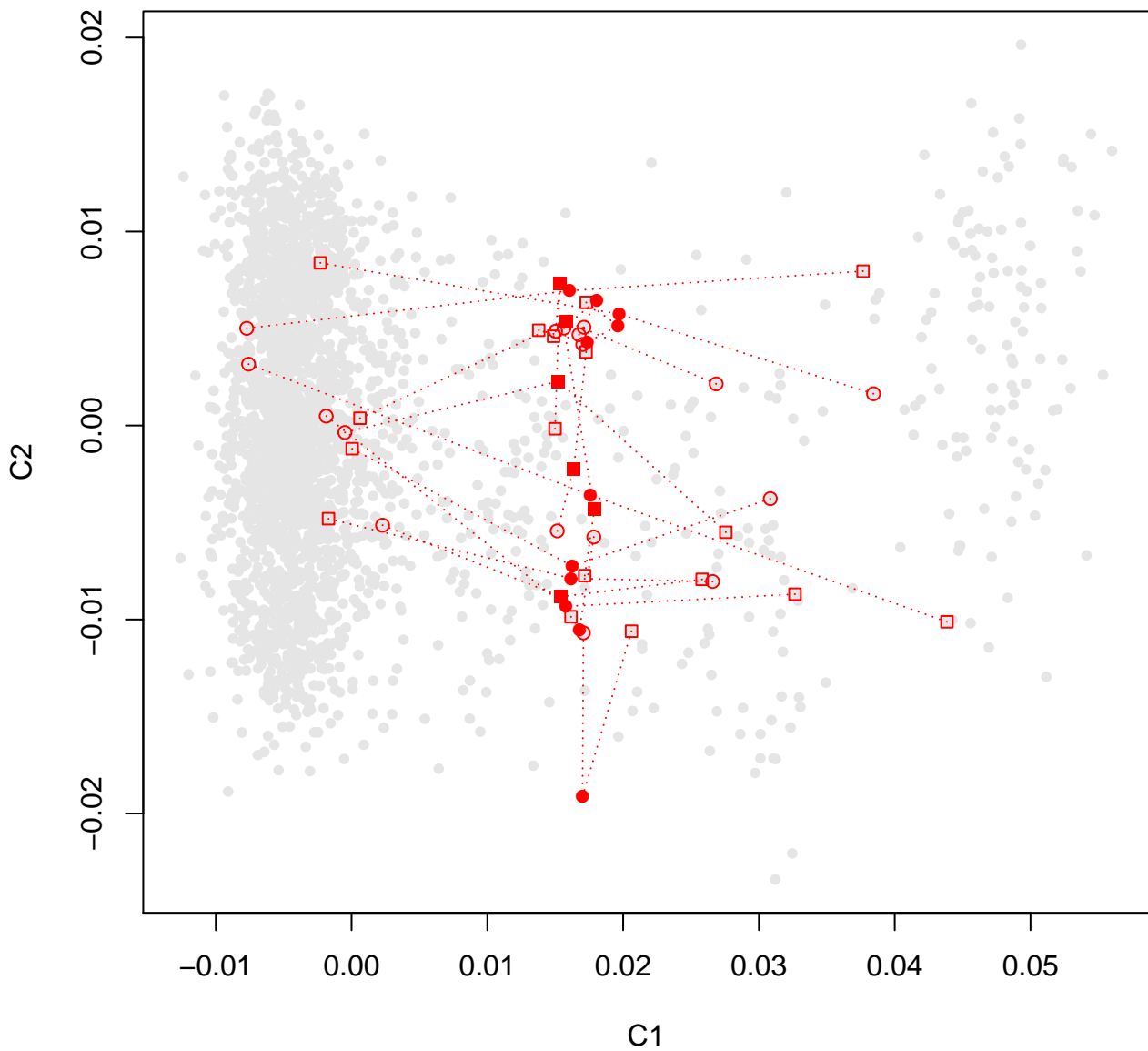

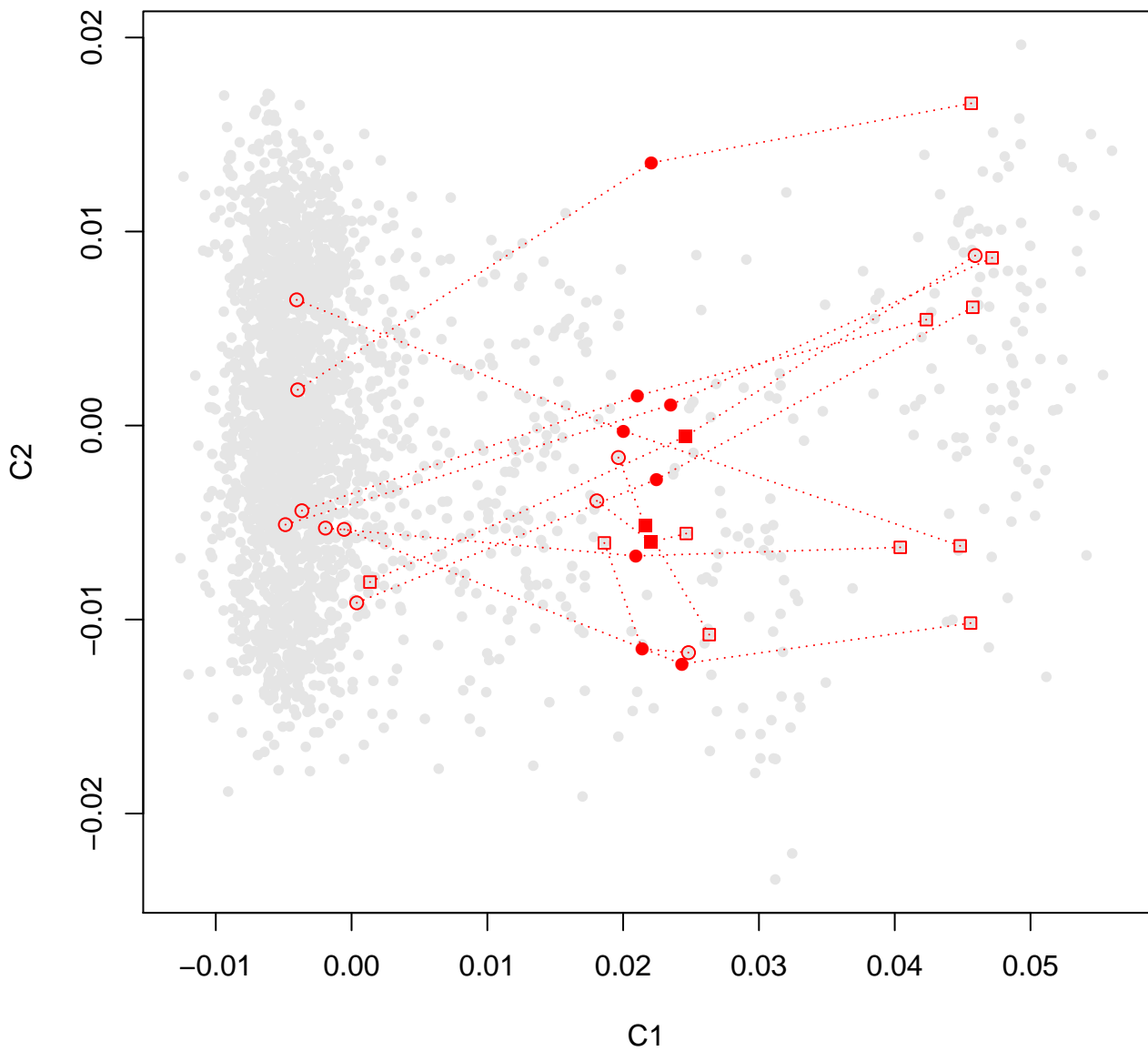

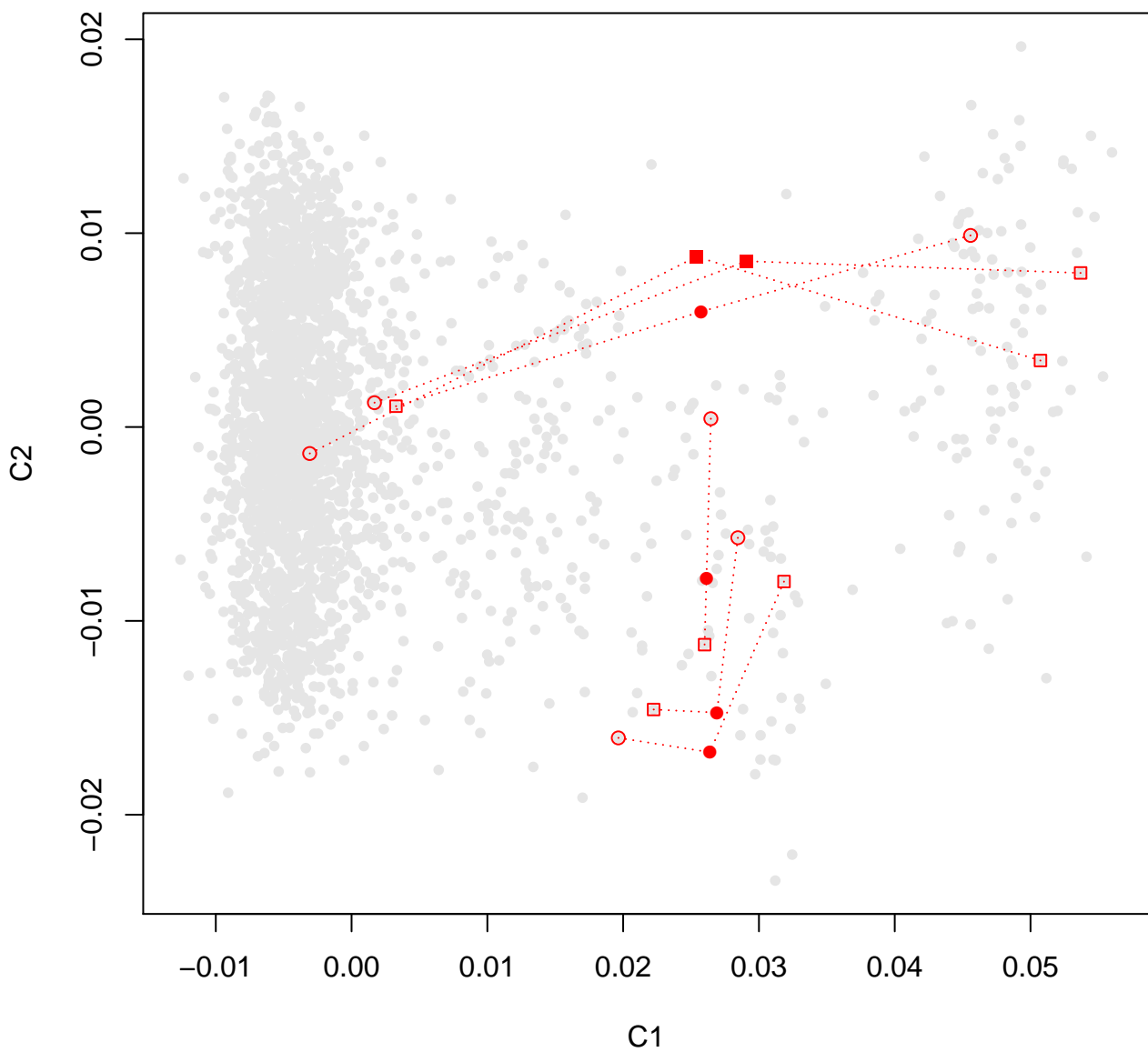

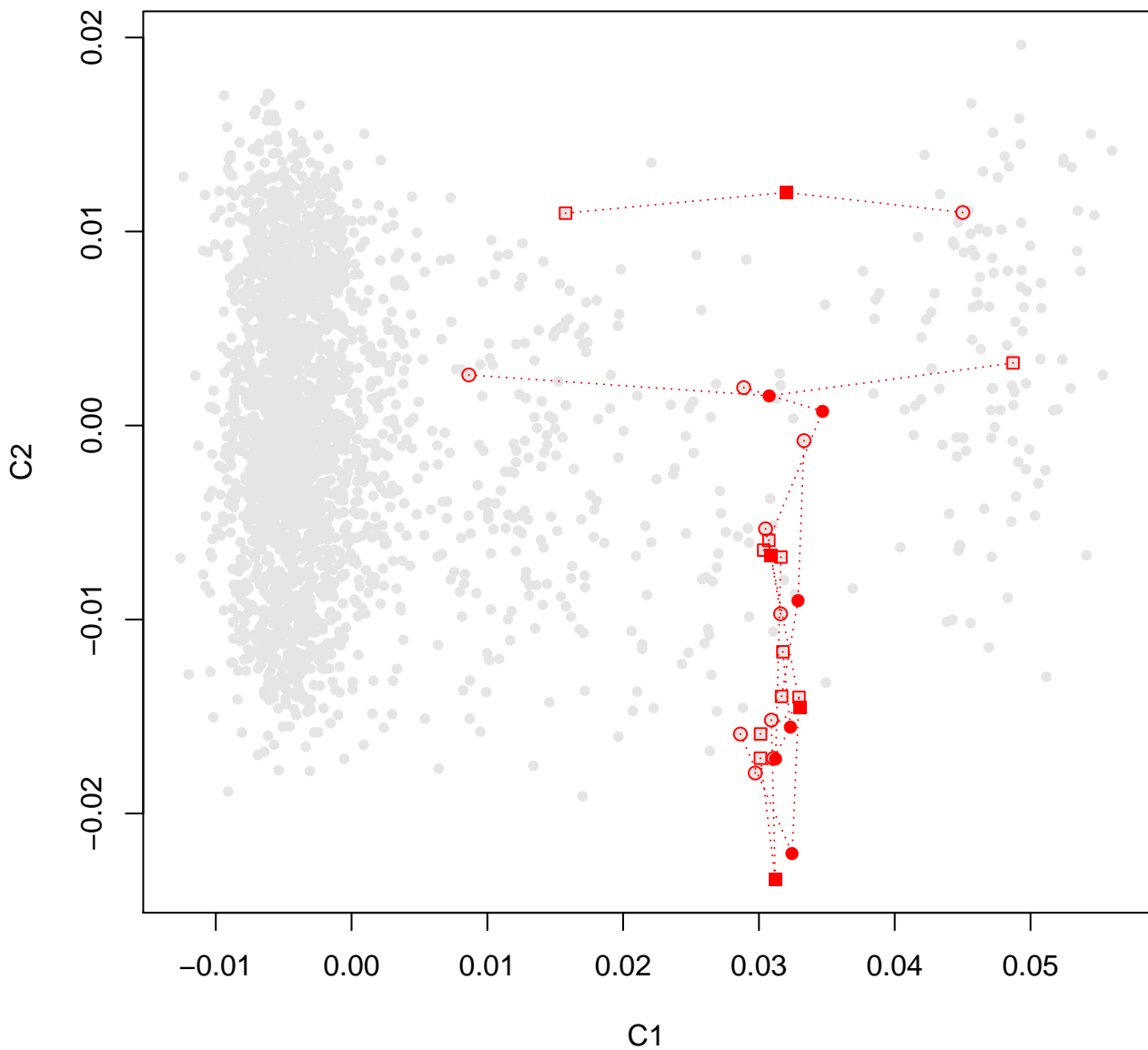

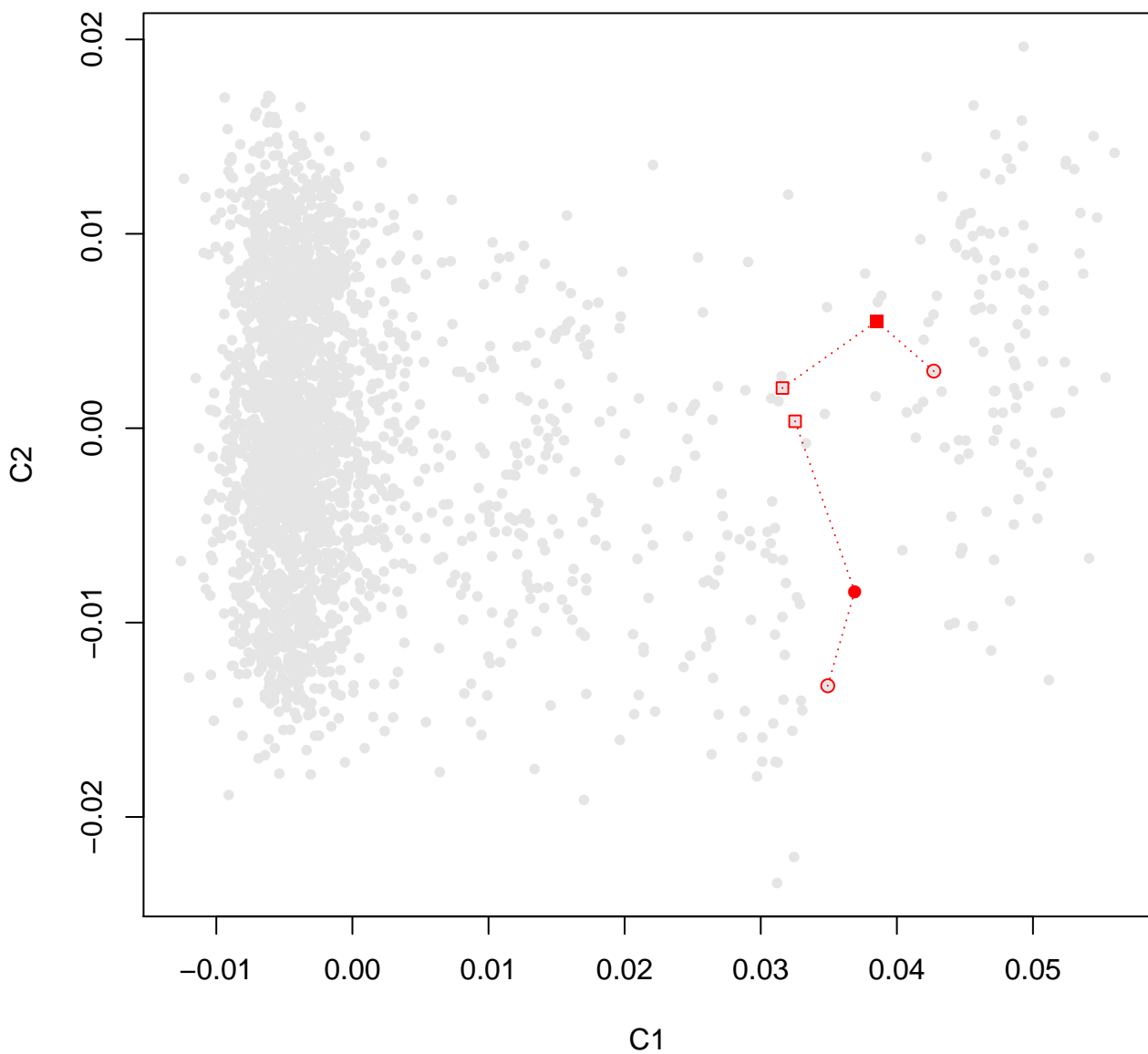

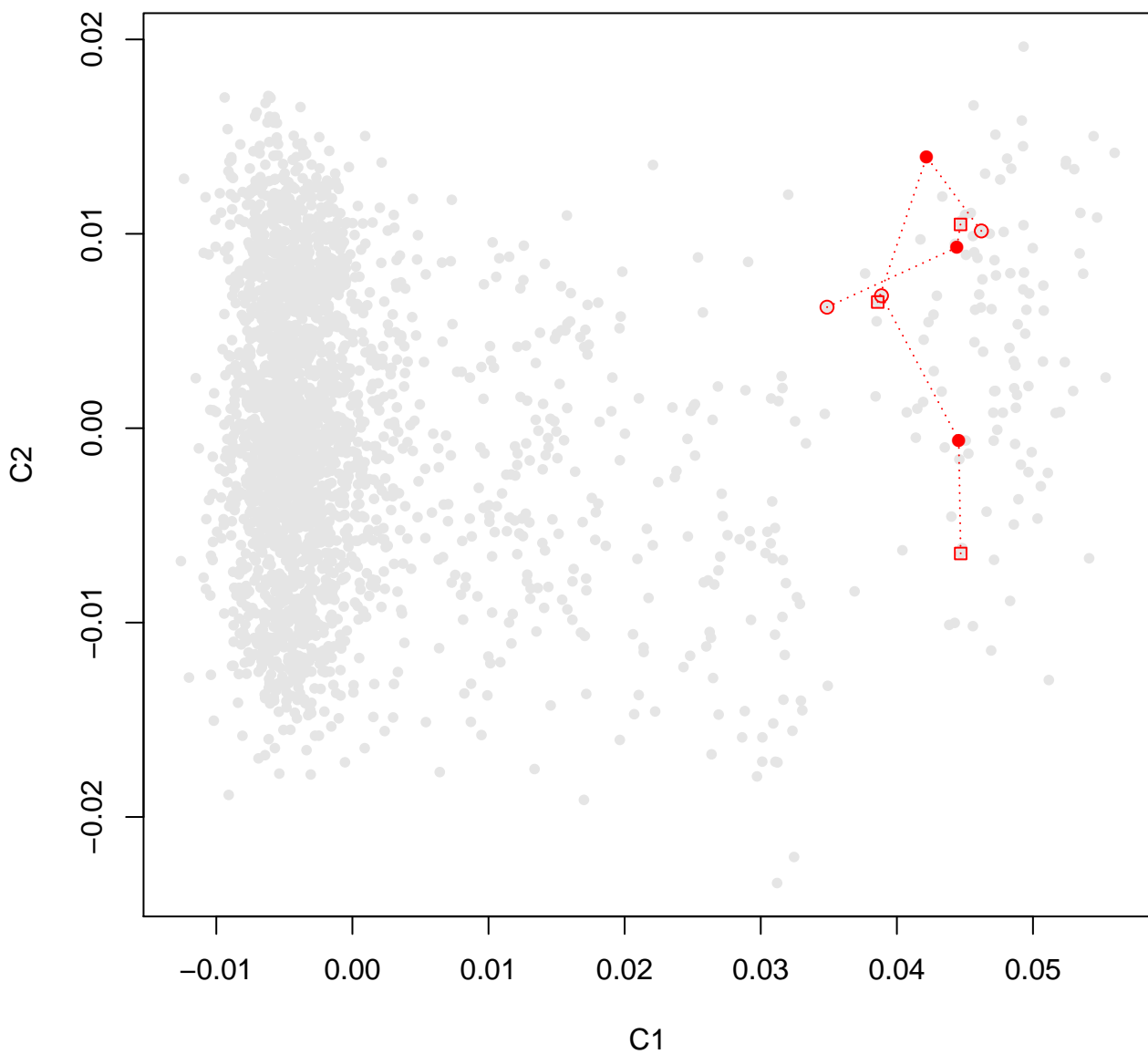

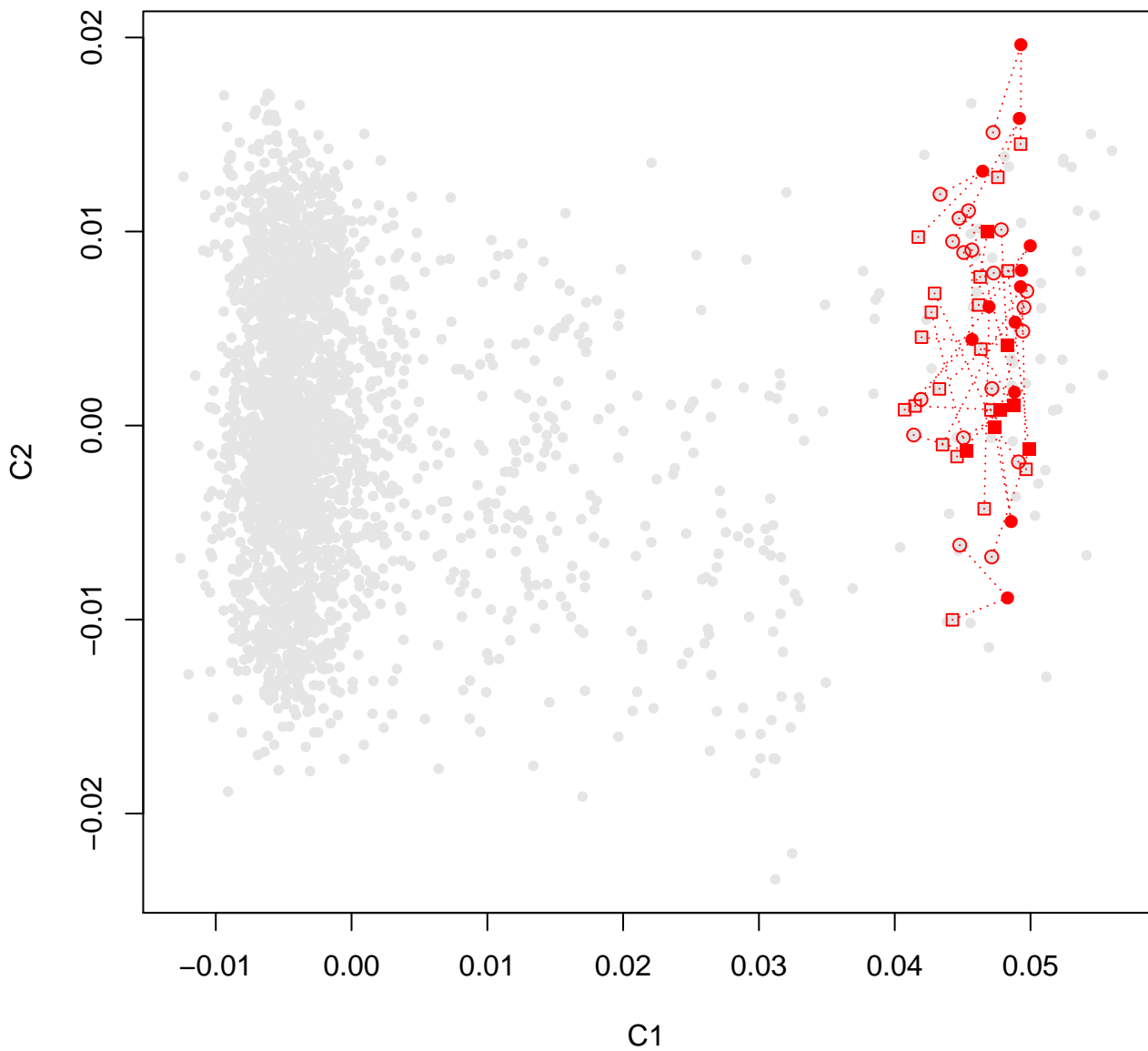

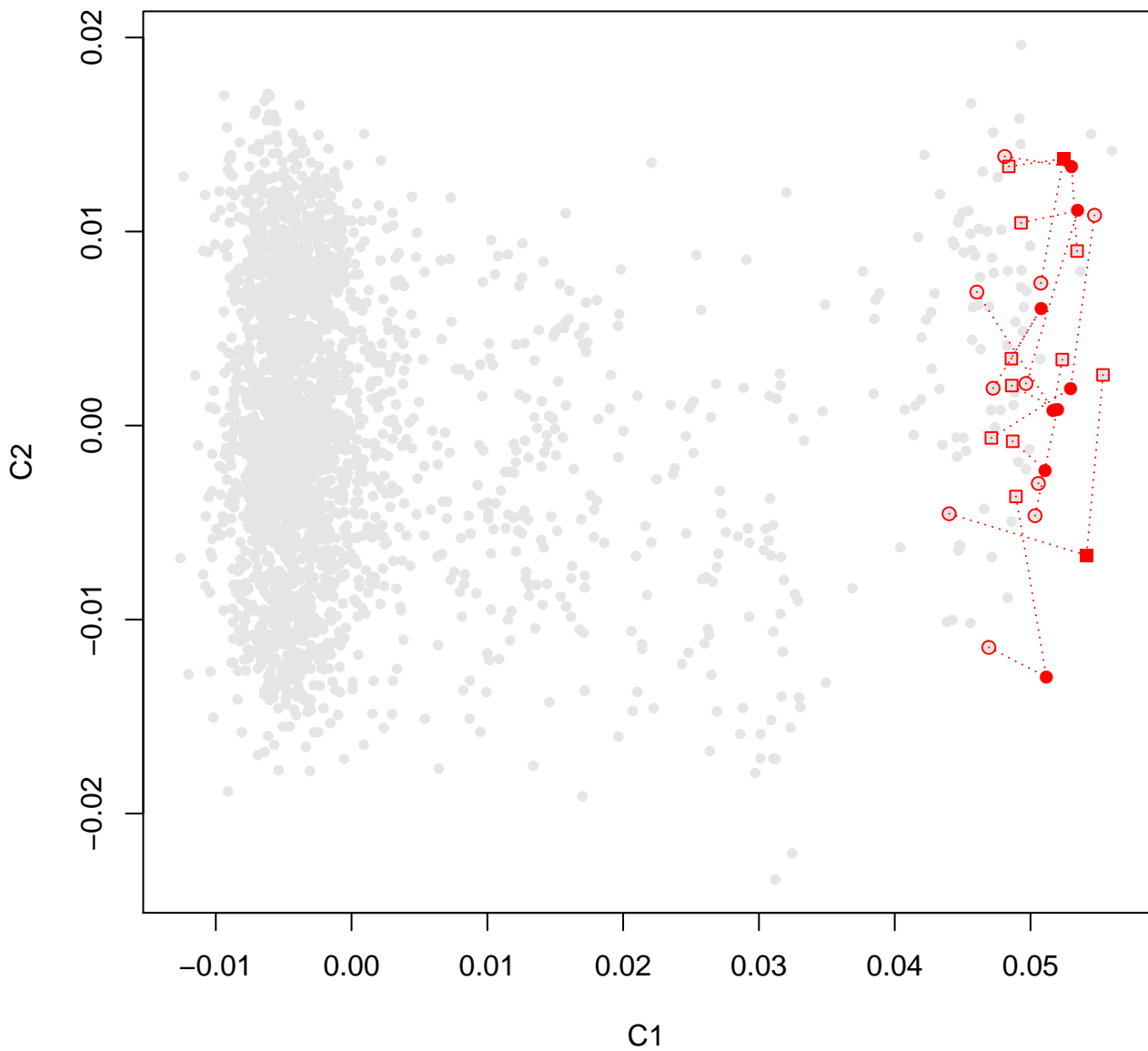

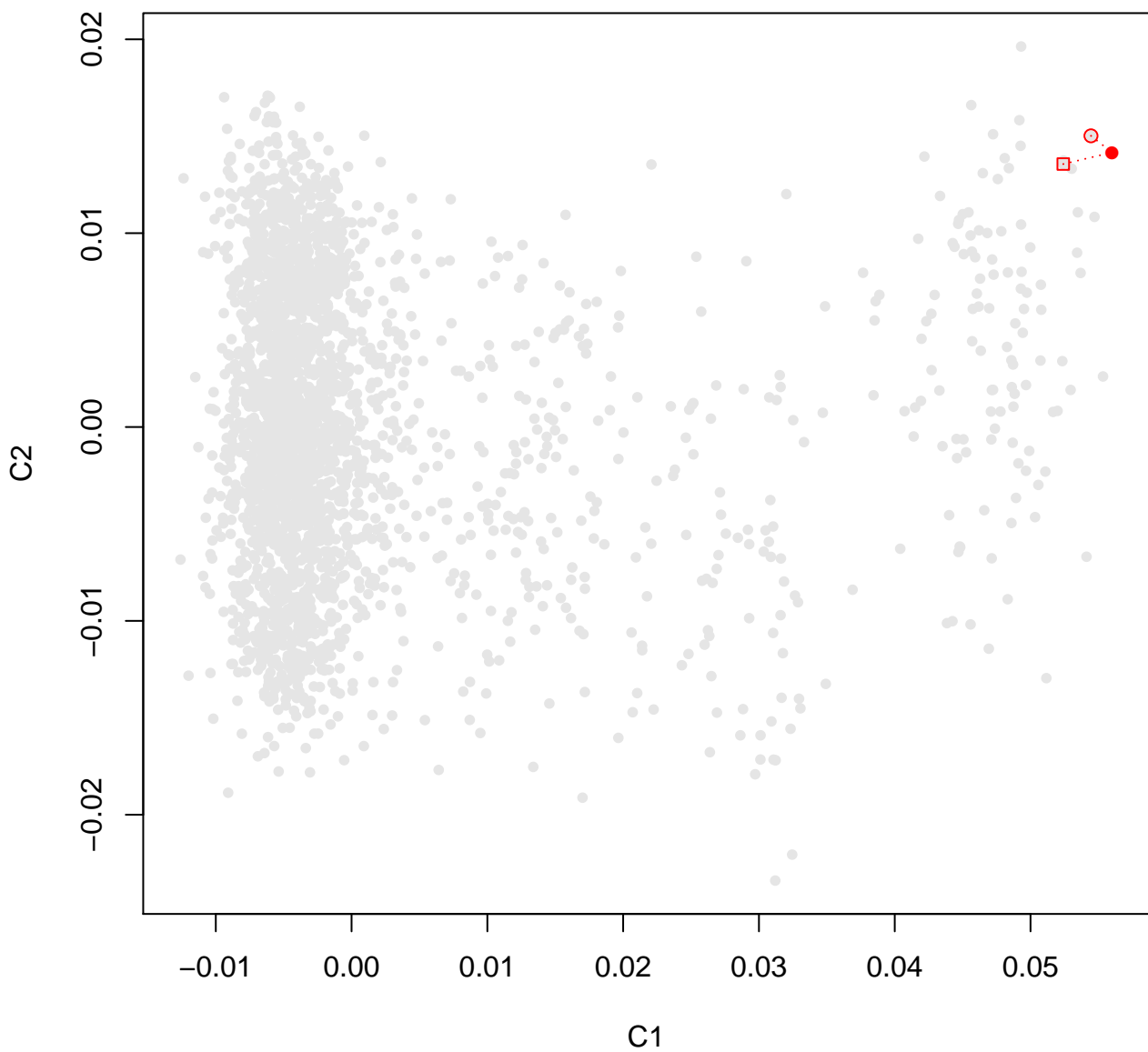

Supplement: Additional file 5: — Supplementary figure PCA of IMSGC. (PDF 230 kb) [file 12881_2015_201_MOESM5_ESM.pdf]
